# CopR, a Global Regulator of Transcription to Maintain Copper Homeostasis in *Pyrococcus furiosus*

**DOI:** 10.3389/fmicb.2020.613532

**Published:** 2021-01-11

**Authors:** Felix Grünberger, Robert Reichelt, Ingrid Waege, Verena Ned, Korbinian Bronner, Marcell Kaljanac, Nina Weber, Zubeir El Ahmad, Lena Knauss, M. Gregor Madej, Christine Ziegler, Dina Grohmann, Winfried Hausner

**Affiliations:** ^1^Institute of Microbiology and Archaea Centre, University of Regensburg, Regensburg, Germany; ^2^Department of Structural Biology, Institute of Biophysics and Physical Biochemistry, University of Regensburg, Regensburg, Germany

**Keywords:** archaea, transcription, Pyrococcus, CopR, copper, regulation

## Abstract

Although copper is in many cases an essential micronutrient for cellular life, higher concentrations are toxic. Therefore, all living cells have developed strategies to maintain copper homeostasis. In this manuscript, we have analyzed the transcriptome-wide response of *Pyrococcus furiosus* to increased copper concentrations and described the essential role of the putative copper-sensing metalloregulator CopR in the detoxification process. To this end, we employed biochemical and biophysical methods to characterize the role of CopR. Additionally, a *copR* knockout strain revealed an amplified sensitivity in comparison to the parental strain towards increased copper levels, which designates an essential role of CopR for copper homeostasis. To learn more about the CopR-regulated gene network, we performed differential gene expression and ChIP-seq analysis under normal and 20 μM copper-shock conditions. By integrating the transcriptome and genome-wide binding data, we found that CopR binds to the upstream regions of many copper-induced genes. Negative-stain transmission electron microscopy and 2D class averaging revealed an octameric assembly formed from a tetramer of dimers for CopR, similar to published crystal structures from the Lrp family. In conclusion, we propose a model for CopR-regulated transcription and highlight the regulatory network that enables *Pyrococcus* to respond to increased copper concentrations.

## Introduction

The archaeal transcription system combines strategies and regulatory mechanisms known from eukaryotic as well as from bacterial species (Werner and Grohmann, 2011; Peeters et al., 2013). Archaea rely on a single RNA polymerase that synthesizes all RNA species in the cell and is highly homologous to the eukaryotic RNA polymerase II (Grohmann and Werner, 2011). The presence of general transcription initiation factors (TATA box binding protein, Transcription factor B, Transcription factor E) and defined promotor elements (B recognition element, TATA box, initially melted region, initiation site) stresses the close relationship to eukaryotes especially for transcription initiation (Blombach et al., 2016). In contrast, the fine-tuning process of gene expression is mainly achieved by bacterial-like transcriptional regulators (Lemmens et al., 2019). Positive or negative regulation is mediated by the binding of these transcription factors (TFs) to promoter regions of specific genes.

The genome of the hyperthermophilic euryarchaeon *Pyrococcus furiosus* contains a total number of 86 putative DNA-binding TFs. However, the exact function of most of these factors, which represent about 4 % of all open reading frames (ORFs), is unknown (Denis et al., 2018). In an attempt to close that knowledge gap, functional and structural aspects of some of these TFs have been analyzed over the last two decades. While the regulation of sugar or sulfur metabolism and other changing environmental conditions have been studied in detail, the underlying mechanisms to maintain metal homeostasis are only poorly understood (Vierke et al., 2003; Lipscomb et al., 2009; Yang et al., 2010; Gindner et al., 2014; Karr, 2014).

Playing an essential role in the cycling of elements, Archaea not only have to transform and make use of a variety of metals but also have to withstand elevated levels in the respective habitat (Bini, 2010). For many organisms, copper (Cu) is one of the essential trace elements used as a cofactor in a variety of proteins. These are mainly involved in electron transfers due to the ability of copper to undergo redox changes from the reduced form Cu^+^ to the oxidized Cu^2+^. Despite its essential role, high intracellular concentrations are toxic for prokaryotic and eukaryotic cells. Copper catalyzes the conversion of H_2_O_2_ to hydroxyl radicals via the Fenton reaction, which leads to oxidative damage of nucleic acids, proteins and lipids (Gunther et al., 1995; Pham et al., 2013). Cu^+^ also is a strong soft metal and can attack and destroy iron-sulfur proteins either by direct interaction or by blocking iron-sulfur cluster biogenesis (Macomber and Imlay, 2009; Tan et al., 2017). To prevent cellular damage, all cells have developed various copper detoxification strategies. In prokaryotes, this is mainly achieved by active export of copper ions and in rarer cases by sequestration or exclusion (Bini, 2010; Martínez-Bussenius et al., 2017).

The two ATPase subfamilies P_1B–1_ (CopA) and P_1B–3_ (CopB) are the key players in cellular copper export. CopA transports Cu^+^, and CopB is proposed to transport Cu^2+^ (Mana-Capelli et al., 2003; Meloni et al., 2014). To elucidate the mechanism of the exporting enzymes, the structures of homologous archaeal Cu-transporting ATPases CopA and CopB were studied in the hyperthermophilic *Archaeoglobus fulgidus*. The two enzymes seem to have different affinities for Cu^+^ and Cu^2+^ (Mandal et al., 2002; Tsuda and Toyoshima, 2009; Agarwal et al., 2010). Recent data, however, suggest that both subclasses, P_1B–1_ and P_1B–3_, have to be assigned as Cu^+^ transporters, which is consistent with the presence of only Cu^+^ in the reducing environment of the cytoplasm (Purohit et al., 2018). Furthermore, a corresponding metallochaperone of the CopZ family is capable of reducing Cu^2+^ to Cu^+^ and is most likely involved in the transport of the reduced ion to CopA (Sazinsky et al., 2007).

Many Archaea use the metallochaperone CopM, which contains a TRASH- instead of a heavy-metal-associated (HMA)-domain of the CopZ family (Ettema et al., 2006). TRASH is a novel domain that has been proposed to be uniquely involved in metal-binding in sensors, transporters and trafficking proteins in prokaryotes (Ettema et al., 2003). In addition to the specific binding of copper by chaperons, copper can also be buffered by small peptides like GSH and other reducing agents, to prevent cellular damage (Rensing and McDevitt, 2013).

In several Archaea, the Cu-transporting ATPase and the copper chaperone are arranged in a conserved copper resistance gene cluster (*cop*), which also contains an additional gene, encoding for a DNA-binding transcriptional regulator. In previous studies, PF0739 has been bioinformatically predicted to be the copper-dependent regulator CopR in *P. furiosus* (Ettema et al., 2006; Villafane et al., 2011; Hong et al., 2019). Based on biochemical data, *in vitro* analysis and growth experiments using knockout strains, CopR was proposed to play opposing regulatory roles in different Archaea: While in *Thermococcus onnurineus* the transcriptional regulator (TON_0836) represses *copA*, both transporter and chaperone are activated in *Saccharolobus solfataricus* (SSO2652) (Villafane et al., 2011; Hong et al., 2019).

Here, we have characterized the metal-sensing transcriptional regulator CopR in *P. furiosus.* First, we described the influence of different metal ions on the DNA-binding ability of CopR to the shared *copR/copA* promoter and analyzed the growth of parental and *copR* knockout strains under increasing copper levels. We further performed a differential gene expression analysis (DGE) and chromatin immunoprecipitation with high-throughput sequencing (ChIP-seq) under normal and copper-shock conditions to elucidate the CopR-regulated gene network in *P. furiosus*. Integrating the genome-wide results with a more in-depth functional and structural characterisation, we propose that CopR acts as a dual regulator to maintain copper homeostasis.

## Materials and Methods

### Strains, Plasmids, and Primers

All strains, plasmids and primers used in the study are listed in Supplementary Table 1.

### Construction of the *copR* Deletion Strain

For the construction of *P. furiosus* parental strain MURPf52 and Δ*copR* strain MURPf74 a modified genetic system was developed for *P. furiosus* DSM3638 allowing markerless disruption of genes onto the chromosome. This system is based on selection via agmatine-auxotrophy and counter selection via 6-methylpurine as described for *P. furiosus* COM1 strain and *T. kodakarensis* (Santangelo et al., 2010; Lipscomb et al., 2011).

First, for disruption of the *Pyrococcus pdaD* gene (PF1623; arginine decarboxylase gene) via a double-crossover event plasmid pMUR264 was constructed according to Kreuzer et al. (2013). The first fusion PCR product containing upstream and downstream regions flanking the *Pf pdaD* gene encoding a Pyruvoyl-dependent arginine decarboxylase was created using the following two primer pairs: (Pf1622_fP_AscI/Pf1622_rP) and (Pf1624_fP/Pf1624_rP_NotI). The second fusion PCR product consisted of a two-gene resistance cassette which was needed for the selection-counter-selection system. The resistance cassette contained a *gdh* promoter, the *hmgCoA* reductase from *T. kodakarensis*, the region coding for the *xgprt* (PF1950, Xanthine-guanine phosphoribosyltransferase) and the histone A1 terminator sequence of *P. furiosus* (Waege et al., 2010). The first part was amplified using the primers: (SimV_NotI_F/SimV_Rv). For the second part, the primer pair: (Pf1624_fP_fus_2/Pf1624_rP_SbfI_N) was used. Both PCR products were combined with single-overlap extension PCR, ligated using a NotI restriction site and inserted into a modified pUC19 vector (Kreuzer et al., 2013) using AscI and SbfI restriction sites.

However, all attempts to markerless delete the *Pf pdaD* gene using this construct were not successful and thus pMUR264 was modified to allow gene disruption via a single crossover event. To remove the second homologous downstream region, plasmid pMUR264 was amplified using the primer pair: (pUC19_SbfI_F/Pf1950_SbfI_R). The resulting PCR product was digested by SbfI and ligated. This plasmid was denoted as pMUR242 and used for transformation of *P. furiosus* as described (Waege et al., 2010; Kreuzer et al., 2013). To obtain the markerless double mutant MUR37Pf, circular plasmid DNA of pMUR242 and strain MURPf27 (Kreuzer et al., 2013) were used and the corresponding transformants were selected with 10 μM simvastatin in SME-starch liquid medium supplemented with 8 mM agmatine sulfate at 85°C for 48 h. Pure cultures of the intermediate mutant MUR37Pf_i were obtained by plating the cells on solidified medium in the presence of 10 μM simvastatin and 8 mM agmatine sulfate. The integration of the plasmid into the genome by single cross-over was verified by analyzing corresponding PCR products.

Cultures of the correct intermediate mutant were washed with medium under anaerobic conditions to remove the simvastatin. In detail, 1.5 ml of a grown culture were centrifuged in an anaerobic chamber for 4 min at 6,000 *g* and resuspended in fresh culture medium without simvastatin. This procedure was repeated three times. For the counter selection, the cultures were grown in the presence of 50 μM 6-methylpurine and 8 mM agmatine sulfate to induce a second homologous recombination step to recycle the selection marker and to eliminate integrated plasmid sequences. Pure cultures were obtained by single cell isolation using an optical tweezer (Huber et al., 1995). Cultures had to be grown in the presence of 8 mM agmatine sulfate and 8 mM Inosine and 8 mM Guanine (I+G). The genotype of the final mutant was confirmed by PCR and Southern blot experiments.

For markerless disruption of the *Pf copR* gene (PF0739), plasmid pMUR527 was constructed. First a modified resistance cassette had to be designed, which was needed for the selection-counter-selection system. The two-gene resistance cassette contained the *Pf pdaD* gene *including* the promoter and terminator region sequence of *P. furiosus* which was amplified using the primers: (PF1623F_Pr_BHI/PF1623R_Term). For the second part, the three primer pairs: (F_PF1950_P_F_Fu/R_PF1950_Prom, F_PF1950_Fs_P/PF1950 _R and F_PF1950_Fus_T/PF1950_T_R_BHI) were used to amplify the promoter, coding and terminator region of the *Pf xgprt gene.* The four PCR products were combined with single-overlap extension PCR and subcloned into pUC19 vector via the SmaI restriction site. In the next step it was cloned via NotI and SbfI restriction sites into plasmid pMUR47 (Kreuzer et al., 2013). The upstream and downstream flanking regions of the *Pf copR* gene were amplified using the primer pairs: (0739upAscIFW/0739up2RW and 0739dofus2FW/0739doNotIRW). Both PCR products were combined with single-overlap extension PCR and cloned into modified pMUR47 vector via the AscI and NotI restriction sites. The resulting construct (pMUR527) was verified by DNA sequencing.

Circular plasmid DNA and strain MURPf37 were used for transformation and selection was carried out in SME-starch liquid medium without agmatine sulfate and I+G at 85°C for 12 h. Pure cultures of the intermediate mutant MUR65Pf_i were obtained by plating the cells on solidified medium. The integration of the plasmid into the genome by single cross-over was verified by analyzing corresponding PCR products.

For the counter selection cells were plated on solidified medium containing 50 μM 6-methylpurine and 8 mM agmatine sulfate to induce a second homologous recombination step to recycle the selection marker and to eliminate integrated plasmid sequences. The genotype of the final mutant (MUR65Pf) was confirmed by PCR and cells had to be grown in the presence of 8 mM agmatine sulfate and 8 mM I+G.

To restore wild type growth properties (growth without agmatine sulfate and I+G) plasmid pMUR310 was created. The newly designed two-gene resistance cassette was amplified from the pUC19 subclone using the primer pair: (pYS_PF1623F_GA/pYS_PF1950R_GA). This PCR product was cloned into PCR-amplified (PF1623_pYSF_GA/PF1950_pYSR_GA) pYS3 plasmid (Waege et al., 2010) using NEB Gibson Assembly^®^ Cloning Kit. Correctness of the construct was tested by Sanger sequencing. 1 μg of the circular plasmid was transformed into MURPf37 and MURPf65 as described (Waege et al., 2010; Kreuzer et al., 2013). Selection was carried out in 1/2 SME liquid medium without agmatine sulfate and I+G at 85°C for 12 h. Pure cultures of the mutant MUR52Pf and MURPf74 were obtained by plating the cells on solidified medium. Plasmid stability was verified by re-transformation into *E. coli* and DNA sequencing of purified plasmids. Final mutants could be grown without agmatine sulfate and I+G supplementation.

### RNAP, TBP, and TFB

For *in vitro* transcription assays and EMSA analysis, we used RNAP purified from *P. furiosus* cells and recombinant TBP and TFB as described previously (Waege et al., 2010; Ochs et al., 2012; Reichelt et al., 2018).

### CopR, CopRΔTRASH, CopRΔHIS, and CopRΔHISΔTRASH

#### Cloning and Expression

The gene sequence of PF0739 was amplified from genomic DNA of *P. furiosus* using primers with additional BamHI and NdeI restriction recognition sites. The PCR product was cloned into vector pET-30b (NEB) using the respective restriction sites. PF0739 protein variants lacking potential metal-sensing domains (ΔTRASH, ΔHIS, ΔHISΔTRASH) were based on the full-length plasmid version and ligated after amplification using one phosphorylated primer, respectively (see Supplementary Table 1). Subsequently, the constructs were transformed into *E. coli* DH5-α for amplification and grown on Kanamycin (50 μg/ml) supplemented LB media. Next, the constructs were transformed into *E. coli* BL21 STAR^TM^ (DE3) expression strain and grown on Kanamycin (50 μg/ml) supplemented LB medium at 37°C. Protein expression was induced by addition of 0.5 mM IPTG to the cell culture medium at an OD_600_ of about 0.6. Cultures were further cultivated at 18°C overnight, before harvesting the cells by centrifugation at 10,000 *g* for 10 min at 4°C. Cells were stored at −80°C until protein purification.

#### Cell Disruption and Pre-purification

For the purification of PF0739 and PF0739 variants, cells were first resuspended in 50 ml low salt buffer containing 40 mM HEPES (pH 7.5), 80 mM ammonium sulfate, 1 mM EDTA, 10% glycerol (w/v) and a protease inhibitor tablet (Roche). The cell lysis was done by sonification on ice, whereby breakage efficiency was monitored at a light microscope. After cell disruption, DNase I (Roche) was added and all cultures incubated at 37°C for 1 hour. In the next step, the lysate was centrifuged at 48,000 *g* for 20 min at 4°C and the supernatant was transferred to a new tube. A pre-purification step was carried out by applying a heat treatment of 90°C for 15 min and subsequent centrifugation at 48,000 *g* for 20 min at 4°C.

#### Affinity and Size Exclusion Chromatography

The supernatant containing the protein of interest was filtered and loaded onto a 5-ml HiTrap^TM^ Heparin HP column equilibrated with low salt buffer. Next, the protein was eluted by gradually increasing the buffer concentration of the high salt buffer (compare low salt, but 1 M ammonium sulfate). Fractions containing PF0739 (checked on SDS-PAGE) were further purified using size exclusion chromatography by pooling and concentrating of the relevant fractions and loading onto a 24-ml HiLoad^TM^ 10/300 GL Superdex^TM^ 200 column pre-equilibrated with low salt buffer. This column was also used to study multimerization of PF0739.

### Growth Experiments Using an Optical Device

*P. furiosus* was cultivated under anaerobic conditions in 40 ml ½ SME medium supplemented with 0.1 % yeast extract, 0.1 % peptone and 40 mM pyruvate at 95°C, as described previously (Fiala and Stetter, 1986; Waege et al., 2010). For growth comparison experiments, the medium was supplemented with different CuSO_4_ concentrations (compare Figure 2) and each condition for MURPf52 (parental strain) and MURPf74 (Δ*copR* strain) was recorded in biological triplicates during 48 hours of incubation by measuring the turbidity changes *in situ* using a photodiode and a LED with 850 nm as light source. The recorded values were converted to cell/ml by using a calibration curve with known cell concentrations, calculated in a Thoma counting chamber (0.02-mm depth; Marienfeld, Lauda-Königshofen, Germany) using phase-contrast microscopy.

### Differential Gene Expression Analysis

#### Growth Conditions and RNA Isolation

*P. furiosus* parental strain MURPf52 was grown in standard medium at 95°C to late-exponential phase. After reaching a cell density of 1 × 10^8^, cells were either shocked by adding 20 μM CuSO_4_ (copper-shock) or left untreated (control) and incubated for 30 min. The experiment was performed in biological triplicates.

Total RNA was isolated using the Monarch RNA purification Kit (NEB), including the recommended genomic DNA removal by on-column DNase treatment, according to the instructions of the manufacturer. Quantity, quality and integrity were measured using Nanodrop One, Qubit RNA HS assay kit (Thermo Fisher Scientific) and the Prokaryote total RNA Nano Kit on a Bioanalyzer to measure RIN values (Agilent).

#### Library Preparation and Sequencing

Library preparation and RNA-seq were carried out as described in the Illumina TruSeq Stranded mRNA Sample Preparation Guide, the Illumina HiSeq 1000 System User Guide (Illumina, Inc., San Diego, CA, United States), and the KAPA Library Quantification Kit - Illumina/ABI Prism User Guide (Kapa Biosystems, Inc., Woburn, MA, United States). In brief, 100 ng of total RNA from *P. furiosus* was fragmented to an average insert size of 200–400 bases using divalent cations under elevated temperature (94°C for 4 min), omitting the mRNA purification step with poly-T oligo-attached magnetic beads. Next, the cleaved RNA fragments were reverse transcribed into first strand cDNA using reverse transcriptase and random hexamer primers. Actinomycin D was added to improve strand specificity by preventing spurious DNA-dependent synthesis. Blunt-ended second strand cDNA was synthesized using DNA Polymerase I, RNase H and dUTP nucleotides. The incorporation of dUTP, in place of dTTP, quenched the second strand synthesis during the later PCR amplification, because the polymerase does not incorporate past this nucleotide. The resulting cDNA fragments were adenylated at the 3’ ends, the indexing adapters were ligated, and subsequently specific cDNA libraries were created by PCR enrichment. The libraries were quantified using the KAPA SYBR FAST ABI Prism Library Quantification Kit. Equimolar amounts of each library were used for cluster generation on the cBot with the Illumina TruSeq SR Cluster Kit v3. The sequencing run was performed on a HiSeq 1000 instrument using the indexed, 50 cycles single-read (SR) protocol and the TruSeq SBS v3 Reagents according to the Illumina HiSeq 1000 System User Guide. Image analysis and base calling resulted in .bcl files, which were converted into FASTQ files with the bcl2fastq v2.18 software.

Library preparation and RNA-seq were performed at the service facility “KFB - Center of Excellence for Fluorescent Bioanalytics” (Regensburg, Germany^1^).

#### Data Analysis Using the DESeq2 Pipeline

For differential gene expression analysis, rRNA-derived Illumina reads were first removed using SortmeRNA (Kopylova et al., 2012). Next, reads in FASTQ format were quality/length/adapter trimmed using trimmomatic (v. 0.36) in single-end-mode (Bolger et al., 2014). Therefore, we allowed for a minimum length of 12 bases and a cut-off Phred score of 20, calculated in a sliding window of 4 bases. We used the STAR aligner (v. 2.5.4) to map the reads to a recently published updated version of the *P. furiosus* genome (Dobin et al., 2013; Grünberger et al., 2019). Mapping statistics are included in the Supplementary Table 2. The sorted BAM files were then used to generate count tables using featureCounts (Liao et al., 2019). Differential gene expression analysis was performed using the DESeq2 pipeline (Love et al., 2014). Furthermore, we used the *apeglm* method for effect size shrinkage and calculation of fold changes (Zhu et al., 2019). All steps of the analysis, including the generation of plots were performed using R and can be found at www.github.com/felixgrunberger/CopR (R Foundation for Statistical Computing, 2018).

Enrichment analysis of archaeal cluster of orthologous genes (arCOGs) was performed by (i) extracting the gene specific arCOG information from the arCOG database^2^ and (ii) performing gene set enrichment using the goseq package, which allows for custom genome sets, calculates enrichment probabilities correcting for transcript lengths and therefore accounts for selection bias (Young et al., 2010; Makarova et al., 2015).

#### Confirmation of Data Using RT-qPCR

RT-qPCR reactions were performed similar as described previously (Reichelt et al., 2018). In short, total isolated RNA was reverse transcribed using the ProtoScript^®^ II First Strand cDNA Synthesis Kit (NEB), according to the manufacturer’s instructions and using a random primer mix (Promega). The reactions were assembled in triplicates using the qPCRBio SyGreen Mix Lo-Rox Kit (PCR Biosystems) with reverse transcribed cDNA from the first step in a 1:10 dilution, including a control reaction that lacked the reverse transcriptase (-RT) and a no template control (NTC). RT-qPCR reactions were run on a Rotor-Gene Q cycler (Qiagen) in a three-step protocol: 95°C – 10’ for one cycle; 95°C – 30”, 58°C – 30”, 72°C – 30” for 40 cycles. Data evaluation was done using the corresponding Rotor-Gene Q software package (Qiagen). Relative expression levels were calculated using the delta-delta Ct method (2^–ΔΔCt^), by comparing the Ct values from biological triplicates of the gene of interest to a house-keeping gene *pf0256*. The applicability of *pf0256* as a calibrator was evaluated before (Reichelt et al., 2018). Accordingly, all primer efficiencies were tested and only primers with efficiencies between 90 and 110% further used in the experiments.

### ChIP-Seq Analysis

#### Immunoprecipitation

We used an adaption of a ChIP-seq protocol that was established previously for *P. furiosus* (Reichelt et al., 2016). *P. furiosus* cells were grown under anaerobic conditions in serum bottles containing 40 ml ½ SME medium at 95°C as described earlier. After the cells reached a density of 2 × 10^8^, formaldehyde was injected into the flask to a final concentration of 0.1 % (v/v). After 60 seconds the crosslinking reaction was stopped by addition of glycine to a final concentration of 15 mM (v/v). For the copper-treated samples, the cells were shocked with 20 μM CuSO_4_ for 5 min before the crosslink reaction was induced.

Cell disruption and DNA fragmentation was performed in one step via sonication for 25 min using the ultrasonic homogenizer Sonopuls HD 2070 (Bandelin, Berlin, Germany) until an average fragment length of 250 to 400 bp. The insoluble particles were removed by centrifugation. For determination of the DNA concentration and fragment length, 1 volume of crude cell extract was mixed with 4 volumes of ChIP elution buffer (10 mM Tris, pH 8.0, 1% (w/v) SDS, 0.1 mM EGTA) and incubated over night at 65°C. After RNase treatment, the DNA was purified using the NucleoSpin^®^ Gel and PCR Clean-up Kit (Macherey-Nagel). The DNA concentration was determined using the Qubit dsDNA BR Assay Kit (Thermo Fisher Scientific) and the fragment length by agarose gel electrophoresis.

For immunoprecipitation (IP) 100 μl Protein G beads (Dynabeads, Invitrogen) were coupled to 3920 μg serum antibodies (total protein) against CopR (PF0739), according to manufacturer’s instructions. Polyclonal antibodies were produced by Davids Biotechnology (Regensburg, Germany) from recombinantly expressed and purified CopR.

100 μl of antibody-beads complexes were mixed with 900 μl of *P. furiosus* crude extract adjusted to a DNA concentration of 4.44 ng/μl (4 μg DNA/sample) in PBST. The samples were incubated with rotation for two hours at room temperature. The immunoprecipitated samples were placed on a magnet, the supernatant was discarded, and the bead-pellet was washed 2× with low salt buffer (50 mM HEPES, pH 7.4, 150 mM NaCl, 1 mM EDTA, 0.1% (w/v) SDS, 0.1% (w/v) Deoxycholic acid, 1% (v/v) Triton X-100), 1× with high salt buffer (50 mM HEPES, pH 7.4, 500 mM NaCl, 1 mM EDTA, 0.1% (w/v) SDS, 0.1% (w/v) Deoxycholic acid, 1% (v/v) Triton X-100), 1× with ChIP wash buffer (10 mM Tris, pH 8.0, 250 mM LiCl, 1 mM EDTA, 0.5% (v/v) Nonidet P-40, 0.5% (w/v) Deoxycholic acid) and 1× with TE buffer (10 mM Tris, pH 8.0, 0.1 mM EDTA) (Aparicio et al., 2005). Each washing step was done with 1 ml buffer by rotation for 1 min. To elute the immuno-bound DNA from the beads, the bead-pellet was resuspended in 25 μl ChIP elution buffer (10 mM Tris, pH 8.0, 1% (w/v) SDS, 0.1 mM EGTA), transferred to a PCR cup and incubated for 10 min at 65°C. The cup was placed on a magnet, the supernatant was transferred to a new cup, the bead-pellet was resuspended in 25 μl TE buffer supplemented with 0.67% SDS (v/v) and incubated for 10 min at room temperature. Afterwards, both eluates were combined in one PCR cup. For the input sample, 400 ng DNA of *P. furiosus* crude extract was mixed 1:4 with ChIP elution buffer.

Eluted complexes and input samples were incubated overnight at 65°C to reverse the crosslink. After the incubation, the samples were treated with RNase A (0.1 mg/ml final concentration) for 15 min at 37°C and Proteinase K (0,2 mg/ml final concentration) for 15 min at 65°C. ChIP-DNA and input DNA were purified using the NucleoSpin^®^ Gel and PCR Clean-up Kit (Macherey-Nagel). The DNA concentration of the input DNA was determined using the Qubit dsDNA BR Assay Kit (Thermo Fisher Scientific).

#### Library Preparation and Sequencing

Library preparations were done using the NEBNext^®^ Ultra^TM^ II DNA Library Prep Kit for Illumina^®^ with the NEBNext^®^ Multiplex Oligos for Illumina^®^ Index Primers Set 2 and 3 according to the manufacturer’s protocol selecting for an approximate insert size of 200 bp. Library quantification was done with the NEBNext^®^ Library Quant Kit for Illumina^®^ according to manufacturer’s instructions. Before sequencing, the libraries were pooled in equimolar ratios. The library pool was quantified with the KAPA SYBR FAST ABI Prism Library Quantification Kit (Kapa Biosystems, Inc., Woburn, MA, United States) and used for cluster generation on the cBot with the Illumina TruSeq SR Cluster Kit v3. Sequencing was performed on a HiSeq 1000 instrument controlled by the HiSeq Control Software (HCS) 2.2.38, using the indexed, 50 cycles single-read (SR) protocol and the TruSeq SBS v3 Reagents according to the Illumina HiSeq 1000 System User Guide. Image analysis and base calling were done by the Real Time Analysis Software (RTA) 1.18.61. The resulting .bcl files were converted into FASTQ files with the CASAVA Software 1.8.2. Sequencing was performed at the service facility “KFB - Center of Excellence for Fluorescent Bioanalytics” (Regensburg, Germany).

#### Analysis of ChIP-Seq Data

FASTQ files were quality/length/adapter trimmed with trimmomatic (v. 0.36) in single-end-mode using a minimum-length of 40 bp, a cut-off Phred score of 20 (Bolger et al., 2014). Reads were mapped to the *P. furiosus* genome using Bowtie 2 (v. 2.2.3) with default settings (Langmead and Salzberg, 2012). SAM files were converted to sorted BAM files using samtools and extended towards the 3’direction to their fragment-size (200 bp) to better represent the precise protein-DNA interaction (Li et al., 2009; Leleu et al., 2010). Position-specific enrichments in BED format were calculated by i) scaling the reads in each dataset according to sequencing depth, ii) calculation of the ratio between IP and input for each replicate, and iii) averaging of the IP/input ratio from the biological triplicates and taking the log_2_ for comparison.

CopR-regulated genes were selected based on i) up-regulation (> 2 fold, adjusted *p*-value < 0.05) of the transcript upon copper shock and ii) CopR ChIP-occupancy of at least 2 in both ChIP-sets (mean values of IP/Input, calculated in a window of 300 bp around gene starts). MEME was used to find a motif in the upstream regions of the selected genes using intergenic regions from *P. furiosus* as a background model and allowing for motif sizes between 8 and 18 (Bailey et al., 2009). To test motif specificity, CopR motif scanning in the upstream region of all annotated TSSs (60 bases window) was performed with FIMO, using all intergenic regions from *P. furiosus* as a background model and applying a multiple testing-corrected *q*-value (FDR rate) threshold of 0.05 (Grant et al., 2011).

#### Confirmation of Data Using Quantitative Real-Time PCR (RT-qPCR)

qPCR primer pairs were designed using the Primer3 software package and quality assessed. qPCR reactions were assembled as technical triplicates using 2× qPCRBioSyGreen Mix separate Rox kit in a total volume of 10 μl. Primers were added to a final concentration of 0.3 μM. 6 μl of the master mix were mixed with 4 μl of template DNA or H_2_O_DEPC_ as NTC. The specificity of the PCR product was verified by melting curve analysis.

qPCR reactions were run on the Rotor Gene Q cycler with a three-step PCR program described in the RNA-seq section. Replicates with a deviation > 0.5 were excluded from the analysis. The fold enrichment was again calculated according to the delta-delta Ct method.

### *In vitro* Assays

#### Electrophoretic Mobility Shift Assay (EMSA)

DNA templates were obtained from genomic DNA by PCR amplification with the corresponding primer pairs (see Supplementary Table 1). One of the two primers was labeled at the 5’-end with a fluorescent dye. 20 nM DNA was assembled in a 15 μl reaction volume containing: 50 ng competitor DNA (Hind-III-digested λ DNA), 670 μM DTT, 20 μg/ml BSA, 6.7 % glycerol, 40 mM HEPES (pH 7.4), 80 mM (NH_4_)_2_SO_4_ and various amounts of proteins and metals, as described in the results part. The reactions were incubated for 5 min at 70°C and analyzed using a non-denaturing 5 % polyacrylamide gel. After electrophoresis, the DNA fragments were visualized with a Fujifilm FLA-5000 fluorescence imager.

#### DNase I Footprinting

DNase I footprinting was performed as previously described (Ochs et al., 2012). In short, the DNA template containing the promoter regions of *pf0739* and *pf0740* was obtained from genomic DNA by PCR amplification (see Supplementary Table 1). HEX-labeled primers were used in two separate reactions for strand-specific labeling. 4.4 nM template DNA was assembled in a 15 μl reaction volume containing: 40 mM Na-HEPES (pH 7.5), 125 mM NaCl, 0.1 mM EDTA, 0.1 mg/ml BSA, 1 mM DTT, 0.5 mM MgCl_2_ and 2.8 μM CopR, 1 μM TBP and 0.8 μM TFB according to Figure 5. After incubation for 20 min at 70°C, 0.05 units of DNase I (Fermentas) was added and incubated for another minute at 70°C. The reaction was terminated by the addition of 5 μl 95 % formamide and incubation for 3 min at 95°C. The DNA was precipitated with ethanol and resuspended in 2–4 μl formamide buffer. A DNA sequencing ladder was generated using a DNA cycle Sequencing Kit (Jena Bioscience) according to manufacturer’s instructions. Samples were loaded onto a 4.5 % denaturing polyacrylamide gel and analyzed using an ABI 377 DNA sequencer.

#### *In vitro* Transcription Assay

*In vitro* transcription assays were performed similar as described previously (Ochs et al., 2012; Reichelt et al., 2018). To this end, template DNAs containing the promoter regions of the respective gene were amplified from genomic DNA or plasmid pUC19/*gdh* by PCR amplification (see Supplementary Table 1). The reactions were assembled in a total volume of 25 μl containing: 2.5 nM template, 5 nM RNAP, 30 nM TFB, 95 nM TBP, 40 mM Na-HEPES (pH 7.4), 250 mM KCl, 2.5 mM MgCl_2_, 5 % (v/v) glycerol, 0.1 mM EDTA, 0.1 mg/ml BSA, 40 μM GTP, 40 μM ATP, 40 μM CTP, 2 μM UTP, and 0.15 MBq (110 TBq/mmol) [α-32P]-UTP, if not indicated otherwise (see Figure 5). After incubation for 10 min at 80°C, the RNA transcripts were extracted by phenol/chloroform, denatured in formamide buffer for 3 min at 95°C and separated on a denaturing 6 % polyacrylamide gel. Finally, phosphor imaging plates were exposed to the sequencing gels and visualization performed using a phosphoimager (FLA-5000, Fuji, Japan).

### Negative-Stain Transmission Electron Microscopy and Image Analysis by 2D Class Averaging

For transmission electron microscopy (TEM), protein solutions with a concentration of 220 ng/μl were chosen. Samples were negatively stained with a solution containing 2 % (w/v) uranyl acetate (UAc) in presence of 0.005 % n-dodecyl β-D-Maltopyranosid (DDM). A carbon film coated grid - 400 Square Mesh (Plano GmbH), was incubated with 3 μL of protein sample for 45 s. Excess stain was blotted off using filter paper and samples washed using 3 μl of a 2 % UAc solution. The blotting and washing procedures were repeated and the sample finally air-dried and stored at room temperature.

Negative-stained grids were imaged on a TEM JEOL-2100F (200 kV) equipped with a 4k × 4k F416 camera with CMOS chip/detector, TVIPS at a 50K magnification (0.211 nm/pixel) with a defocus range from −0.5 to −1.4 μm. The contrast transfer function for a total of 32 micrographs was determined with CTFfind4 (Rohou and Grigorieff, 2015). Subsequently, the particles were extracted with a mask of 180 Å and processed in RELION 3.0 (Zivanov et al., 2018) to yield the 2D class averages.

## Results

### *Pyrococcus copR* Is Part of the Conserved Archaeal *cop* Gene Cluster

**FIGURE 1:**
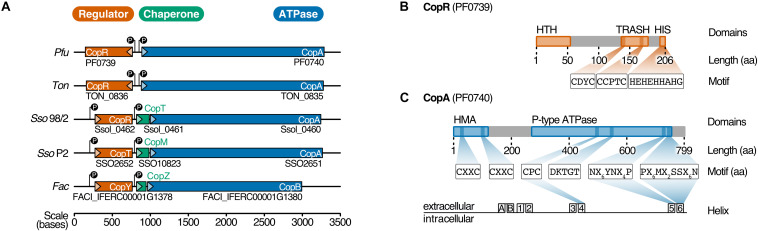
PF0739 (CopR) is part of the conserved archaeal cop cluster in *Pyrococcus furiosus*. **(A)** Copper regulation in Archaea is achieved by a highly conserved *cop* gene cluster consisting of a transcriptional regulator (CopR/CopT/CopY, red), a transporter (CopA/CopB, blue) and optionally a chaperone (CopT/CopM/CopZ, green). The organization of the *cop* cluster in *P. furiosus* was compared to *cop* clusters of other archaeal organisms (*Ton* = *Thermococcus onnurineus*, *Sso96/2 = Saccharolobus solfataricus 96/2, SsoP2 = Saccharolobus solfataricus P2, Fac = Ferroplasma acidarmanus*) (Baker-Austin et al., 2005; Ettema et al., 2006; Villafane et al., 2011; Hong et al., 2019). Genes are drawn to scale; directionality is indicated by arrows. **(B)** Schematic representation of the transcriptional regulator (CopR) encoded by *pf0739* in *P. furiosus*. The regulator consists of a N-terminal helix-turn-helix (HTH) domain that mediates DNA binding, and a metal-sensing TRASH domain (Ettema et al., 2003). The C-terminal Histidine-rich sequence (HIS) is only found in *Thermococcales*. Further sequence features of the domains are highlighted. **(C)** Bioinformatical analysis of conserved amino acids in transmembrane helices 4, 5 and 6 classify PF0740 as the copper exporter CopA.

A conserved *cop* resistance gene cluster plays a critical role in copper homeostasis in Archaea. The gene cluster has been identified in various archaeal species using comparative genomics (Ettema et al., 2006). This *cop* cluster consists of a copper-exporting P_1B_-ATPase CopA (*Ferroplasma acidarmanus* Fer1: CopB), a transcriptional regulator CopR (*Saccharolobus solfataricus* P2: CopT, *F. acidarmanus* Fer1: CopY) and occasionally the metallochaperone CopT (*S. solfataricus* P2: CopM, *F. acidarmanus* Fer1: CopZ) (Figure 1A; Baker-Austin et al., 2005; Ettema et al., 2006; Villafane et al., 2009; Hong et al., 2019).

**FIGURE 2:**
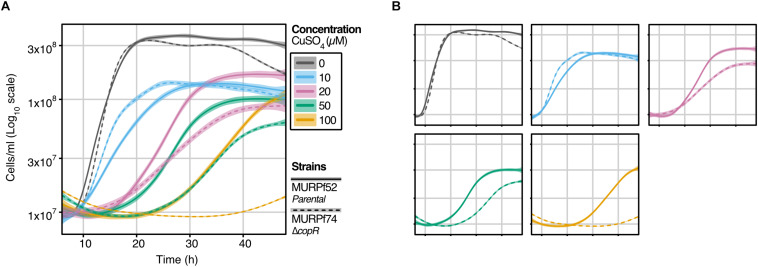
Growth analysis of the *P. furiosus* parental strain (MURPf52) and CopR-knockout strain (MURPf74) in the presence of CuSO_4_. Triplicates of 40 ml cultures were analyzed in standard medium at 95°C, supplemented with 0 to 100 μM CuSO_4_ (indicated by color scale). Growth was recorded during 48 hours of cultivation of the parental strain (solid lanes) and the *copR* deletion strain (dashed lines). Each curve represents the fitted line of three independent experiment, with the shaded area displaying the confidence interval (0.99). **(A)** To compare the effect of the increasing copper concentrations curves are represented in a single graph, while in panel **(B)**, strain-dependent effects are highlighted.

In *Pyrococcus furiosus*, the transcriptional regulator CopR is encoded by the gene *pf0739*, which is in divergent orientation to *copA* (*pf0740*). It consists of an N-terminal helix-turn-helix domain and a C-terminal metal-sensing TRASH domain together with a Histidine-rich region (HIS), the latter one is only present in the order *Thermococcales* (Figure 1B; Ettema et al., 2003). To unravel more details about the function of CopR in *P. furiosus*, we expressed the wild type protein together with three mutants in *Escherichia coli* and tested the proteins for DNA binding using gel-shift assays. Here, the intergenic region between *copR* and *copA* served as target DNA. Binding of CopR to the target region with increasing protein concentrations resulted in a specific protein-DNA shift (Supplementary Figure 1). The mutated variants, lacking the putative metal-binding domains, TRASH, HIS or both, showed a very similar DNA binding affinity for all variants, indicating that DNA binding is primarily mediated by the HTH domain whereas the metal-binding domains are dispensable for DNA binding.

To determine both selectivity and sensitivity towards the recognized metal of the CopR/CopA system in *P. furiosus*, we performed two types of experiments: First, motif analysis of the heavy metal-binding domains (HMBDs) classified PF0740 as a copper-exporting ATPase of type 1B (Figure 1C; Argüello, 2003; Sitsel et al., 2015). Secondly, increasing concentrations of different metal ions (AgNO_3_, CuSO_4_, FeCl_3_ and, CoCl_2_) were supplemented in the binding assays of CopR. All ions reduced the binding affinity, but the most potent effect of the analyzed metals was observed for copper and silver ions (Supplementary Figure 2A). In contrast to the copper-induced release in the EMSA analysis using the full-length CopR, the effect was slightly reduced in the case of a CopRΔHIS mutant and significantly reduced for a CopRΔTRASH mutant (Supplementary Figures 2B–D). It is interesting to note that lower metal concentrations resulted in a smear with reduced mobility and higher levels in an increasing amount of released DNA. The minimal concentration used in the binding assays that resulted in CopR release was 12.5 μM CuSO_4_, which is very similar to the detection range of CopR in *T. onnurineus* (79 % amino acid sequence identity) (Hong et al., 2019). Taken together, these results indicate that the CopR/CopA system is involved in copper regulation in *P. furiosus*.

### Deletion of copR Transcriptional Regulator Leads to a Copper-Sensitive Phenotype

To learn about the importance of CopR for copper-detoxification in *P. furiosus*, a *copR* deletion mutant was constructed, using an established genetic system in this hyperthermophilic organism (Waege et al., 2010; Kreuzer et al., 2013). Growth analysis of this deletion mutant (MURPf74) in comparison to the parental strain (MURPf52) was performed using increasing amounts of copper (0 to 100 μM). Starting at sub-lethal concentrations, we observed prolonged lag phases and reduced cell densities in both strains (Figure 2).

While the collected curves were almost identical for 0 and 10 μM CuSO_4_, higher copper concentrations caused a significant effect on the growth of the knockout strain. In contrast to the parental strain, the growth of the *copR*-disrupted strain was almost completely abolished in the presence of 100 μM copper. This finding supports the idea that copper homeostasis is a tightly controlled system with a sensitivity in the μM-range and indicates that CopR acts as a transcriptional activator of the copper-exporting ATPase *pf0740* in *P. furiosus*.

### Characteristics of the *P. furiosus* Transcriptome in Response to a Copper-Shock

**FIGURE 3:**
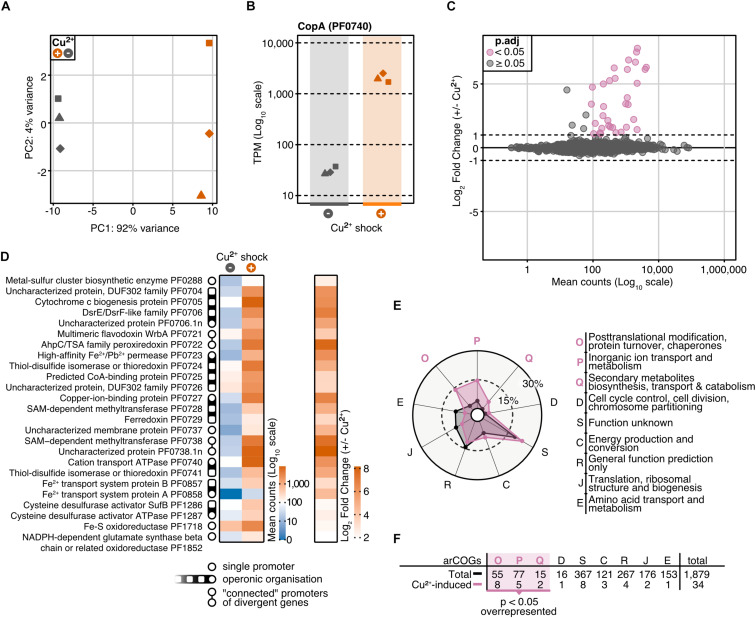
Differential gene expression analysis of *P. furiosus* after 20 min copper shock with 20 μM CuSO_4_. **(A)** Principal component analysis of variance stabilized transformed RNA-Seq read-counts of normal conditions (dark blue) and Cu^2+^ shock conditions (red) shows that most of the variance in the experimental setup is caused by the treatment of the cells. Replicates are indicated by different shapes. **(B)** Comparison of raw RNA-Seq read-counts mapping to PF0740 shows a 6.14 Log_2_ Fold Change after Cu^2+^ shock (adjusted *p*-value = 1.80e–173). **(C)** MA plot showing the distribution of mean read counts (log_10_ scale) against log_2_ fold change. 34 genes that are more than 2-fold up-regulated (with an adjusted *p*-value < 0.05) are highlighted in pink. **(D)** Heatmaps for mean read-counts for control and Cu^2+^ shock condition are shown for the 25 most significant regulated genes. Promoter and operon organization are indicated in the legend. **(E)** Enrichment analysis of archaeal clusters of orthologous genes (arCOGs) found in the 34 significantly up-regulated genes (compare panel **C**). Contribution of each category is calculated in percentage and compared to the total background set with significantly up-regulated categories marked in pink. **(F)** Enrichment analysis is based on the comparison of the total number of genes found in an arCOG category (Total) and the number of genes that are significantly up-regulated (>2 fold).

To investigate the role of CopR in the copper regulation network in *P. furiosus*, we applied an integrative approach, combining differential gene expression (DGE) analysis and genome-wide binding analysis by ChIP-seq. For DGE, we cultivated the parental strain (MURPf52) until the middle of the log phase, shocked the cells with 20 μM CuSO_4_ for 20 min and isolated the RNA for next-generation sequencing. PCA confirmed that indeed the copper-shock (and not handling of the biological replicates) caused most of the variance in the experimental setup (Figure 3A). By analyzing the transcript abundances, we could confirm the essential role of *copA* in removing excess ions from the cell, observing a 70-fold up-regulation of the mRNA levels after copper treatment (Figure 3B).

Altogether, 34 genes were more than 2-fold up-regulated, but not a single gene was down-regulated to the same extent (adjusted *p*-value cut-off: 0.05) (Figures 3C,D). Furthermore, transcription of *copR* was only slightly increased (1.5-fold, Supplementary Table 3), which is in agreement with the regulatory role instead of being part of the detoxification system. The possible role as an activator is also in agreement with RT-qPCR experiments which confirmed the up-regulation of two of the most prominent genes (PF0740, PF0738.1n) in the wild type strain, but not in the knockout strain in the presence of Cu ions (Supplementary Figure 3). Notably, *pf0727* is among the most up-regulated genes (105-fold). Based on the domain annotation and strong induction upon copper treatment, PF0727 is most likely the missing chaperone in the *cop* cluster in *P. furiosus*. Due to the presence of an HMA domain instead of a TRASH domain the protein belongs to the CopZ and not to the CopM family. A closer look at the clusters of archaeal orthologous genes (arCOGs) revealed that most of the up-regulated genes belong to the groups O (posttranslational modification, protein turnover, chaperones), S (function unknown) and P (inorganic ion transport and metabolism) (Figures 3E,F; Makarova et al., 2015). The group of the hypothetical genes consists of eight candidates, including *pf0738.1n*, which exhibits the most substantial up-regulation (290-fold). A phylogenetic analysis revealed that most of these genes are only conserved within the Thermococcales and not in other archaeal CopR-based copper resistance systems (Supplementary Figure 4).

### Integrative RNA-Seq and ChIP-Seq Identifies CopR Targets

**FIGURE 4:**
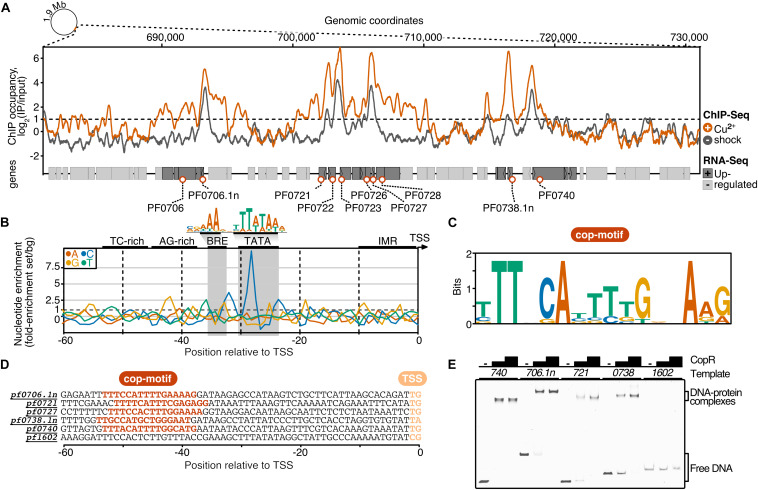
ChIP-seq and integration with differential gene expression data identifies CopR as a global regulator of copper homeostasis in *P. furiosus.* **(A)** ChIP occupancy of CopR zoomed to genomic region ∼680,000 to 730,000, which contains the 14 most up-regulated genes from the differential gene expression analysis (compare Figure 3). ChIP-seq curves were generated for Cu^2+^ shocked (orange) and untreated (gray) samples by comparing the IPs to input samples (mean values of triplicates are shown). Genome annotation is shown at the bottom according to scale with significantly up-regulated genes (adjusted *p*-value < 0.05, Log_2_ fold change +/- Cu^2+^ > 1) colored in dark gray. Up-regulated genes that are bound by CopR under both conditions (IP/Input > 1) are highlighted with orange circles. An example of an unbound genomic region is shown in Supplementary Figure 5A). **(B)** Nucleotide enrichment analysis of upstream regions dependent on the transcription start sites comparing the sequences of genes fulfilling the selection criteria (orange circles, *n* = 10) with the nucleotide content of sequences contributing to the consensus motif of primary transcripts in *P. furiosus*. The consensus motif, which consist of a B recognition element (BRE) and a TATA box, is highlighted above the enrichment plot (Grünberger et al., 2019). **(C)** MEME motif analysis of selected genes. A semi-palindromic motif positioned directly upstream of the BRE element was found. **(D)** Promoter sequences of genes that were further analyzed by EMSA or ChIP-qPCR (compare Supplementary Figure 5B) highlighting the cop-motif in orange. **(E)** EMSA analysis (20 nM DNA, 0/200/400 nM protein) of selected promoter regions confirms specific binding of CopR to multiple regions, whereas no binding to a control promoter (*pf1602, gdh*) could be observed.

It is interesting to note that the 14 most up-regulated genes are located within a 28 kb region of the genome. To answer the question if the transcriptional activation of these genes upon intoxication is connected to the binding of CopR, we performed a ChIP-seq experiment with and without copper shock (20 μM CuSO_4_). The results from these experiments demonstrated a very similar relative CopR occupancy independent of the copper treatment (Figure 4A). This finding is in agreement with the EMSA analysis, where a considerable amount of the transcriptional regulator remained bound to the DNA after addition of 25 μM CuSO_4_ (comparable amount as used in the *in vivo* experiments, see Supplementary Figure 2). Furthermore, the binding pattern of CopR overlaps with the upstream regions of up-regulated genes or operons under both conditions, which confirms specific binding of CopR, as well as the possible role in transcriptional activation. Next, we defined a set of 10 CopR-regulated promoters by selecting genes that are i) up-regulated under copper conditions (*p*.adj < 0.05, fold change > 2) and ii) whose promoters have CopR bound under copper-free and copper-shock conditions *in vivo* (mean occupancy IP/input 300 bp around gene start > 2).

**FIGURE 5:**
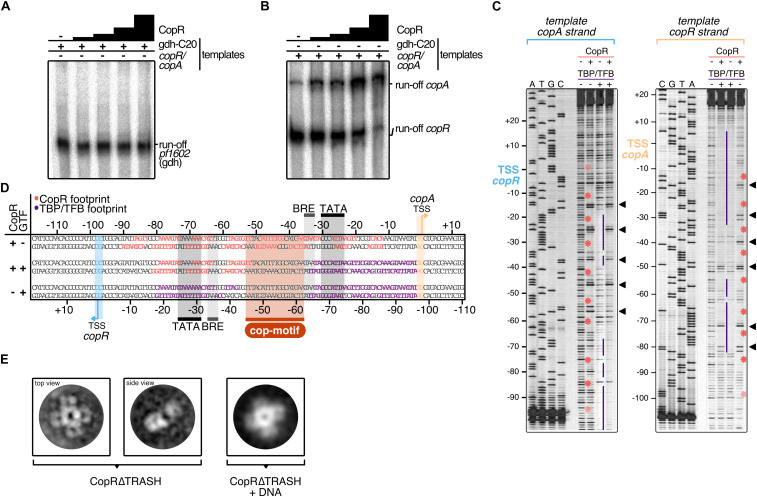
Mechanistic and structural characterization of CopR. **(A)** Influence of CopR on *in vitro* transcription. 2.5 nM of the *gdh* and **(B)** the *copR/copA* templates were transcribed in the presence of increasing concentrations of CopR (0.3, 0.6, 1.2, 2.3 μM). **(C)** DNase I footprint on the *copR/copA* template in the presence of CopR, TBP/TFB and all three components. TBP/TFB-protected regions (purple lines), CopR-protected regions (red asterisks) and hypersensitive sites (black triangles) are highlighted. **(D)** Summary of the protected regions determined in the *in vitro* DNAse I footprinting assay. Promoter elements, transcript boundaries and the semi-palindromic CopR recognition motif are highlighted. **(E)** Representative 2D class averages of CopRΔTRASH in the absence and presence of a *copR/copA* DNA template reveal an octameric assembly formed by a tetramer of dimers in both states.

To elucidate the sequence specificity of CopR binding and regulation, we compared the nucleotide content of the CopR-regulated promoter regions to a background set consisting of 763 sequences that contributed to a recently published consensus motif in *P. furiosus* (Grünberger et al., 2019; Figure 4B). This archaeal-typical promoter motif is characterized by elements that facilitate transcription by the recruitment of the basal transcription factors TFB (BRE element) and TBP (TATA box) and melting of the region initially upstream of the transcription start site (TSS). In comparison to the *Pyrococcus* consensus motif, the promoter sequences of the up-regulated genes showed some minor deviations in the BRE element and the conserved A(T) at position −10 that contributes to the promoter strength (Torarinsson et al., 2005), but the most striking difference was a C-enriched TATA box (Figure 4B). Further upstream of the promoter sequence we identified a TC-rich and AG-rich signature from −35 to −50 that also differed from the consensus sequence content. A motif enrichment analysis using MEME identified a semi-palindromic-like motif with the minimal palindromic consensus sequence TTNNCAWWWTGNNAA, which is located at almost all CopR-regulated promoters directly upstream of the BRE element (8 of 9 with an annotated TSS) (Figures 4C,D). Scanning of this motif in the promoter region of all known TSSs using FIMO further confirmed the specificity of regulation, as all of the motif occurrences can be assigned to genes that are bound and up-regulated by CopR (false discovery rate < 0.05) (Grant et al., 2011).

To validate the findings of the ChIP-seq experiments, we performed gel-shift assays and ChIP-qPCR, that both confirmed specific binding of CopR to multiple genomic regions (Figure 4E) and enrichment under both conditions (Supplementary Figure 5B).

Based on the genome-wide binding pattern and in combination with the results from the DGE analysis, we propose a currently undescribed regulating role of CopR on a global level to maintain copper homeostasis in *P. furiosus*.

### CopR Activates Transcription *in vitro*

To verify the stimulating role of CopR, we performed *in vitro* transcription experiments with a DNA template that allows simultaneous transcription of the divergently orientated *copR* and *copA* genes (Figures 5A,B). In the absence of CopR, the main transcript originated from the own strong promoter, and *copA* was only weakly transcribed. However, with increasing concentrations of CopR, transcriptional output increased for the *copA* gene and *copR* transcription was significantly reduced (Figure 5B). In contrast, it did not affect a control template, lacking the CopR binding site (Figure 5A), which clearly indicates that CopR is responsible for both, activation of *copA* and repression of *copR*. The reason for the observed *in vitro* CopR-induced activation is not known, since the stimulating effect of *copA in vivo* was observed only under the presence of copper ions. Attempts to increase the *copA* stimulating effect by adding CuSO_4_ or AgNO_3_ failed. The presence of such ions inhibited the transcription in general (data not shown).

For additional information about the mechanism of activation, we performed DNase I footprinting experiments at the *copR/copA* promoter (Figures 5C,D). The binding of CopR revealed an extended binding pattern in this region consisting of eight strong single footprints in the central region of the fragment and some additional weaker footprints towards the border of the fragment. Most of the strong signals are separated by hypersensitive sites separated by approximately one helical turn (Figure 5C). The correlation of the binding motif obtained from the ChIP-seq experiment with the footprint pattern revealed that the motif is located between the divergent TBP/TFB binding sites of *copR* and *copA.* CopR footprint signals are positioned in the center of the motif and nearby upstream and downstream. These signals are separated by hypersensitive sites, which touch only one or two bases of the beginning or the end of the consensus sequence. Simultaneous presence of the two basal transcription factors, TBP and TFB, and CopR in the reaction (Figure 5D, lane 2), revealed that under these conditions only CopR is in contact with DNA in the region of the *copR* promoter. This finding is in agreement with the identified repression of *copR* in the *in vitro* transcription experiments. In contrast, the position of the CopR footprint upstream of the TBP/TFB footprint that is still present at the *copA* promoter enables CopR-mediated stimulation of *copA*. Similar to the *in vitro* transcription assays, we were not able to supplement the reactions with CuSO_4_ to get further mechanistic insights in CopR regulation. Hong et al. recently encountered the same technical limitation during their description of CopR from *T. onnurineus*. They hypothesized that high magnesium concentrations in the transcription buffers possibly prevent other metal inducing effects (Hong et al., 2019). Notably, the upstream regions of the divergent CopR/CopA genes in *P. furiosus* and *T. onnurineus* have a very high sequence similarity and share almost identical promoter and CopR-recognition sites (Supplementary Figure 6).

### Toward a Structural View of CopR

CopR is the crucial player in the copper-triggered differential regulation of genes that supports detoxification of the cell. At the same time, however, it is not apparent how activation is achieved considering that CopR always seems to be bound to the respective promoter regions. Therefore, we aimed to elucidate the structural properties of CopR. To this end, we developed a purification protocol for the isolation of highly pure CopR. Based on the comparison of CopR to standard calibration proteins, elution profiles of size exclusion chromatography runs (Superdex 200) indicated an octameric conformation (Supplementary Figure 7). As the elution profile revealed an increased symmetric peak for CopRΔTRASH in comparison to the wild type protein, we have selected CopRΔTRASH for further analysis.

Negative-stain TEM imaging confirmed an extremely high monodisperse fraction of the CopRΔTRASH mutant. Finally, 2D classification of more than 50,000 particles, gave rise to the assumption that CopR forms an octameric assembly oriented in a cruciform-like structure (Figure 5E). Due to the high similarity of the structure to LrpA and F11 (Leonard et al., 2001; Yokoyama et al., 2007), we assume a similar outside orientation of the helix-turn-helix domain of the four CopR dimers that positions DNA at the outside of the dimer. Furthermore, CopR complexed with DNA of the *pf706.1n* promoter did not alter the overall octameric structure of the protein (Figure 5E). With a diameter of 16 nm, a DNA fragment of about 150 bp would be necessary to completely wrap the DNA around the protein (Leonard et al., 2001).

## Discussion

From the combination of *in vitro* based approaches, genetic manipulation and the integration of DGE and ChIP-seq data used in this study, we conclude that CopR from *P. furiosus* is a copper-sensing global regulator of transcription and essential during copper detoxification.

The importance of CopR for maintaining copper homeostasis became apparent by growth experiments. While we could show that a *P. furiosus* parental strain can grow under μM-concentrations of Cu^2+^ (up to 100 μM were tested), the growth defect on a CopR-knockout strain was significant. We assume that limiting amounts of essential components necessary for maintaining copper homeostasis are responsible for the observed growth phenotypes. The most obvious component is CopA, which showed a 70-fold increase at the RNA level under copper-shock conditions. *In vitro* experiments confirmed that enhanced *copA* transcription is mediated by CopR-induced activation. The *substantial copA* enrichment is also in agreement with the observed CopR-binding to the upstream region of *copA* as indicated by EMSA and DNase I footprinting analysis. In summary, we assume that CopR is responsible for sensing copper concentrations and transcriptional activation of the corresponding genes necessary to maintain copper homeostasis.

Our findings for CopR are in agreement with data from *S. solfataricus* strain 98/2 and *Halobacterium salinarum* as the corresponding knockout mutants also revealed CopR as a positive regulator for *copA* transcription (Kaur et al., 2006; Villafane et al., 2011; Darnell et al., 2017). In contrast, recent data from the closely related *T. onnurineus* NA1 suggested CopR as a repressor for autoregulation and *copA* transcription (Hong et al., 2019). This fundamental discrepancy for *copA* transcription is remarkable in the light of the almost identical divergent organization of the upstream regions of *copR* and *copA* and 79% sequence identity between both CopR proteins instead of 30% to the *Saccharolobus* CopR. A comparison in more detail revealed that in *Pyrococcus* -in contrast to *Thermococcus*- low μM CuSO_4_ concentrations do not lead to a full release of the protein from the DNA but result in a low mobility complex indicating a conformational change of the CopR-DNA complex. These findings are also in line with *in vitro* DNA-binding studies from *S. solfataricus* strain P2, which also suggest a partial rearrangement of CopR-binding in the presence of copper instead of dissociation (Ettema et al., 2006).

An additional difference between both organisms is the quaternary structure of CopR in solution: In the case of *Thermococcus* a tetrameric structure was determined using size exclusion chromatography analysis (Hong et al., 2019) and for *Pyrococcus* an octameric complex was found by negative-stain TEM imaging and gel filtration experiments. In order to explain these differences, it is tempting to speculate that the presence of a His_6_ tag in the N-terminal region of *Thermococcus* CopR is responsible for the observed different quaternary structure of the protein. Furthermore, it is possible that the presence of this tag also contributes to an increased sensitivity to copper towards dissociation from the DNA instead of allowing a conformational switch necessary for transcriptional activation. To verify the different regulation mechanism of CopR in *T. onnurineus* NA1, additional information about the susceptibility of the mentioned *copR* deletion strain (Hong et al., 2019) to increasing copper concentrations or the behavior of CopR without a His_6_ tag would be helpful.

Despite the inconsistency of the function of CopR as repressor or activator for *copA* transcription between *Thermococcus* and *Pyrococcus*, the ChIP-seq data collected in this study demonstrate an almost identical DNA binding pattern of CopR independent of the presence or absence of copper ions. This finding also points to a required structural rearrangement of CopR on the DNA to activate transcription in the presence of copper ions. Such behavior would be very similar to the function of the copper-sensing transcription factor CueR from *Escherichia coli* (Philips et al., 2015). CueR can activate transcription by controlling open complex formation, while it is continuously bound to DNA (Martell et al., 2015). This mechanism may permit more rapid responses to environmental changes. In an evolutionary context, the high copper-toxicity could have been a driving force for the independent development of this regulatory mechanism in different domains. Nevertheless, there is no sequence similarity between both proteins, CueR belongs to the predominant bacterial MerR family of regulators (PROSITE documentation PDOC00477), and CopR belongs to the Lrp/AsnC family (PDOC00520). The latter one is a rather old family of prokaryotic transcriptional regulators and very common in Archaea (Peeters and Charlier, 2010). Crystal structures of several archaeal Lrp members indicate a highly conserved octameric structure with an N-terminal winged HTH motif for DNA binding and a C-terminal domain necessary for oligomerization and effector binding (Leonard et al., 2001; Yokoyama et al., 2007; Kumarevel et al., 2008). Our TEM imaging data of negative-stained CopR also revealed a tetrameric assembly of dimers with most likely the DNA wrapped around the protein. This finding is in line with published structures of DNA-protein complexes FL-11 and Grp (Yokoyama et al., 2007; Kumarevel et al., 2008; Yamada et al., 2009) which further confirmed an accumulated occurrence of an octameric assembly within the Lrp family. The identification of extended DNA binding regions and hypersensitive sites in footprinting experiments is also in agreement with the assumed wrapping of the DNA around the octamer (Ouhammouch, 2001; Liu et al., 2014). Therefore, the behavior of CopR is very similar to published data of the Lrp family with the difference that CopR uses copper ions as effector instead of molecules of the amino acid metabolism. However, the ability to use a variety of effector molecules is pervasive for the archaeal subfamily. A detailed analysis of eight Lrp/AsnC paralogs in *Halobacterium salinarum* revealed that these proteins are involved in regulating genes in response to copper or oxidative stress, changes in K^+^ or NAD^+^ concentrations or modified growth conditions (Plaisier et al., 2014).

The Lrp/AsnC family is not only involved in the regulative response to a wide range of different physiological conditions but also employ different mechanisms of transcriptional regulation. Repression by preventing the recruitment of the RNAP is demonstrated for LrpA from *P. furiosus* (Dahlke, 2002) and activation by stimulating the binding of TBP is shown for Ptr2 in *Methancaldococcus jannaschii* (Ouhammouch et al., 2003). Besides, a dual regulator mechanism has been shown for Ss-LrpB from *Saccharolobus solfataricus*, which activates transcription at low factor-concentrations, whereas at high concentrations, transcription is repressed (Peeters et al., 2009).

Based on our results, we conclude that CopR also has a dual function as repressor and activator, but the situation seems to differ from the LrpB from *S. solfataricus*. *In vitro* transcription experiments in the absence of CopR indicate a strong *copR* promoter without the necessity for further activation. However, *in vivo*, CopR remains always bound to the *copR* promoter region, which blocks TBP/TFB recruitment, represses transcription of its gene and only allows some basal expression. This goes in line with the DGE data, which indicate low-level expression of *copR* independent of copper ions and high-level expression of *copA* in the presence of copper. Similar results about these differences in the expression rates were also described in *Saccharolobus solfataricus* P2 (Ettema et al., 2006), *Saccharolobus solfataricus* 98/2 (Villafane et al., 2009), *Sulfolobus metallicus* (Orell et al., 2013), *Ferroplasma acidarmanus* Fer1 (Baker-Austin et al., 2005) and *Halobacterium salinarum* (Kaur et al., 2006).

**FIGURE 6:**
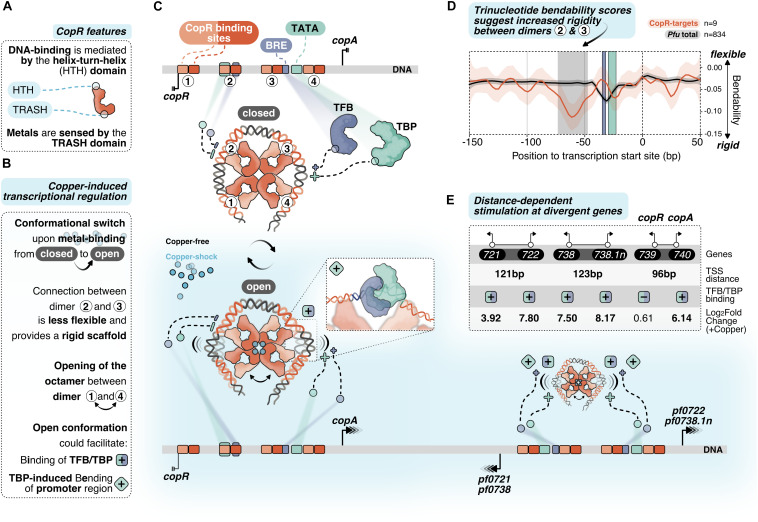
Putative model of allosteric CopR regulation in *P. furiosus.* **(A)** Domain architecture of CopR (monomer shown schematically in red) highlighting the DNA-binding HTH domain and the metal-sensing TRASH domain. **(B)** Binding of copper presumably triggers a conformational switch from a closed state to a complex, which is opened between dimer 1 and 4. In combination with the rigid connection between dimers 2 and 3 it is possible that this conformational switch could facilitate TBP/TFB binding (+ square) and/or TBP-induced bending to the corresponding promoter regions (+ turned square). **(C)** CopR-regulated promoter regions include CopR-binding sites (orange/red) and the archaeal-specific promoter elements BRE (recruits TFB, purple) and the TATA box (bound by TBP, light-green). Transcription start sites (TSS) are indicated by vertical lines. Transcription is either repressed (3 lines) or stimulated (arrows) under copper-shock conditions (lower panel, light-blue). The octameric CopR assembly in open conformation allows bending of critical promoter regions and facilitates binding of TBP/TFB depending on the distance of the divergent TSS: While CopR prevents binding of general transcription factors to the *copR* promoter, TBP/TFB can bind to the second promoter (*copA*). Simultaneous binding of CopR and two sets of GTFs stimulates transcription in both directions for *pf0722/pf0271* and *pf0738/pf0738.1n*. **(D)** Major groove bendability of selected promoters revealed increased rigidity between dimers 2 and 3. Major groove bendability of promoter sequences was estimated based on trinucleotide scales derived from DNase-I cutting frequencies (Brukner et al., 1995; Meysman et al., 2014). More negative values are the result of less cutting by DNase-I and indicate that the DNA is not bend towards the major groove and is therefore less flexible. Trinucleotides were extracted from CopR-target promoters (*n* = 9) and all available promoters defined in *P. furiosus* previously (Grünberger et al., 2019) from –150 bp to +50 from the TSS. Each line represents the smoothed conditional mean with confidence intervals (0.95) displayed as shaded areas. Boxes represent area between dimers 2 and 3 (gray), BRE (purple) and TATA box (light-green). **(E)** Summary of the TSS-distance dependent stimulation of divergent CopR-regulated transcripts.

To interpret our results and to integrate these data with the knowledge gained for the Lrp family in general, we suggest the following regulation mechanism for divergent transcription (Figure 6): Under normal growth conditions, CopR binds to multiple binding sites with about 150 bp of DNA wrapped around each octamer. Each dimer of the octamer is in “direct contact” with a weakly conserved DNA sequence as indicated by motif analysis of the ChIP-seq data and the footprinting experiments. We assume an increased affinity to binding sequences located directly upstream of promoter sequences and cooperative binding, which seems to be a common feature of Lrp molecules (Peeters et al., 2004; Chen et al., 2005), to weaker signals stimulated by the octameric structure of CopR downstream of the promoter. An additional contact of Lrp-like molecules downstream of the TATA box in combination with transcriptional activation is already described for Ptr2 and BarR (Ouhammouch et al., 2005; Liu et al., 2014).

For transcriptional activation, we suggest an allosteric regulation mechanism where the binding of effector molecules (most likely Cu^+^) is sensed by the TRASH domain alone or in combination with the HIS stretch at the C-terminal end (Figure 6A). The role of the TRASH domain in copper binding is indicated by the decreased metal sensitivity of the ΔTRASH mutant in the gel shift assays (Supplementary Figure 2D) and was also previously demonstrated by mutational analysis in *T. onnurineus* NA1 (Hong et al., 2019).

Due to the binding of the metal, we hypothesize a conformational switch resulting in the opening of the quaternary structure of the octamer similar to the structure of Lrp from *Escherichia coli* or FL11 with bound arginine from *Pyrococcus* OT3 (de los Rios and Perona, 2007; Yamada et al., 2009). We assume that opening between dimer 1 and 4 is preferred due to increased flexibility at this position as these dimers are not directly linked with the wrapped DNA (Figures 6B,C). In contrast, there is a direct connection from dimer 1 to dimer 2, 3 and 4, which most likely provides a more rigid scaffold. The bendability of CopR-regulated promoters in comparison to the total set of promoters in *P. furiosus* (Grünberger et al., 2019) was estimated by comparing trinucleotide scores (Brukner et al., 1995; Meysman et al., 2014) and clearly shows a less flexible region between dimer 2 and 3 (Figure 6D). The movement of dimer 1 towards 2 and dimer 4 towards 3, initiated by the opening of the octamer, may reduce torsional stress on the DNA between these corresponding pairs of dimers, which could either facilitate the accessibility of TBP and TFB to the corresponding promoter sequences or enable TBP-induced bendability or is involved in both (Figures 6B,C). Torsional stress on the DNA as limitation for binding of TBP was already demonstrated by single molecule FRET experiments in *Methanocaldococcus jannaschii* (Nickels et al., 2016).

Interestingly, provided a divergent gene organization and an appropriate distance between the two TSSs, one CopR octamer can promote transcriptional activation of two separate gene clusters (Figure 6E). Simultaneous stimulation by CopR requires a distance of about 122 bp between the two TSSs (*pf0721/pf0722*; *pf0738/pf0738*.1n). In contrast, the reduced distance of 96 bp in the case of the *copR*/*copA* gene cluster leads to repression of the *copR* gene independent of the presence of copper ions, most likely due to binding interference of dimer 3 with the *copR* promoter. Additionally, there is also the possibility of activation at only one side of the octamer, whereas the position of the other side is located at the end of another gene (*pf0726*/*pf0727*).

**FIGURE 7:**
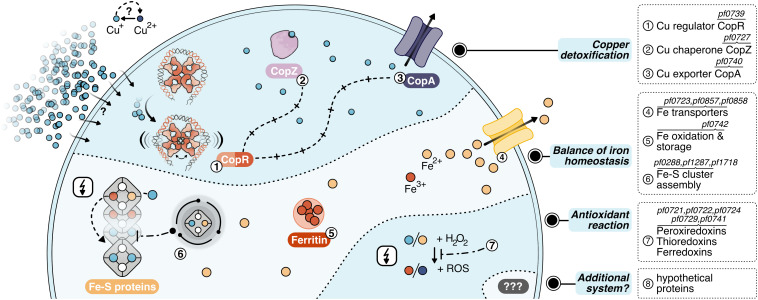
Layers of copper detoxification in *P. furiosus*: In a primary response, CopR (1, orange-red) senses excessive amounts of Cu^+^ ions (light-blue), that entered the cell by a currently unknown mechanism or diffusion and activates the transcription of the copper-chaperone CopZ (2, light-purple) and the copper-exporter CopA (3, purple). After CopZ potentially delivers the Cu^+^ ions to the transporter, they are exported out of the cell. The main toxic effect of copper ions is caused by replacing iron ions in iron-sulfur clusters. Therefore, the induction of Fe-S cluster assembly proteins, Fe-transporters and ferritin helps to re-balance iron homeostasis (4–6). Additionally, antioxidant enzymes prevent the induction of the Fenton reaction (7), which otherwise causes toxicity by the production of reactive oxidative species (ROS).

Besides mechanistic details of CopR transcriptional regulation, our data also allow insights into a general copper-specific transcriptomic response (Figure 7). To avoid a transcriptomic response due to cell-death rather than a metal-specific response, we applied a moderate copper shock using 20 μM CuSO_4_ in all *in vivo* experiments. However, under these conditions, we already saw a strong phenotype of the CopR knockout mutant, which emphasizes the essential role of CopR. Using this “semi-toxic” concentration in the DGE analysis, we found 34 strongly up-regulated genes (>2-fold) upon copper-shock (Supplementary Table 3). The transcriptional pattern under copper shock conditions is comparable to other metal stress transcriptomic responses in prokaryotes and eukaryotes, and apart from metal-specific genes also includes non-metal related genes that cooperatively contribute to metal resistance (Bini, 2010; Lagorce et al., 2012).

For the primary detoxification mechanism, *Pyrococcus* relies on the induction of the ATPase CopA and the metallochaperone CopZ (PF0727), which directly interact with the copper ions. An additional cluster of genes deals with i) iron homeostasis involving several transporters to pump iron ions (PF0723, PF0857, PF0858) (Zhu et al., 2013), ii) ferritin (PF0742) which combines oxidation of Fe^2+^ to Fe^3+^ together with storage of the oxidized iron inside the protein cavity (Honarmand Ebrahimi et al., 2015) and iii) Fe-S cluster assembly proteins (PF0288, PF1286, PF1287, PF1718). This collection of genes fit well into the recently emerging concept that the primary toxic effect of copper is the replacement of iron in iron-sulfur cluster proteins and not the conversion of H_2_O_2_ to hydroxyl radicals (Lagorce et al., 2012; Tan et al., 2017). Therefore, the induction of these proteins helps to re-balance displaced iron ions and to avoid inactivation of iron-sulfur proteins. Besides, antioxidant enzymes as peroxiredoxins, thioredoxins or ferredoxins (PF0721, PF0722, PF0724, PF0729, PF0741) can also assist in preventing the induction of the Fenton reaction by the released Fe^2+^ or Cu^+^ ions. This is in line with the finding that some of these enzymes are also induced after exposure to hydrogen peroxide (Strand et al., 2010). Since the constitutively expressed superoxide reductase also produces hydrogen peroxide (Thorgersen et al., 2012; Khatibi et al., 2017), it is most likely that the induction of these antioxidant enzymes successfully inhibits the production of hydroxyl radicals via the Fenton reaction under this low dose of copper. In consequence, there is almost no induction of genes dealing with general stress response or DNA repair mechanisms. In contrast, a copper shock in *Metallosphaera sedula* induced a mixed gene population of metal-specific and also generic responses indicating that the conditions used have had much more potent effects concerning viability in comparison to our setup (Wheaton et al., 2016).

In this context, it is interesting to note that *Metallosphaera* uses an additional mechanism for copper resistance: sequestration with inorganic polyphosphate to facilitate export from the cytoplasm (Rivero et al., 2018). Such a mechanism is also described for *S. solfataricus*, as a mutant strain -unable to accumulate polyphosphate-showed an increased copper sensitivity in spite of *copA* up-regulation (Soto et al., 2019). Based on our data, there is no indication that a comparable system is implemented in *Pyrococcus*. Nevertheless, the induction of eight hypothetical proteins opens up the possibility that different sequestration systems or an additional mechanism for copper detoxification exist within the Thermococcales.

## Author’s Note

This manuscript has been released as a pre-print at bioRxiv (Grünberger et al., 2020).

## Data Availability Statement

The R scripts detailing the analysis can be found in the corresponding Github repository under www.github.com/felixgrunberger/CopR. Raw sequence data have been uploaded to the SRA and are available under project accession number PRJNA603674.

## Author Contributions

FG did the DGE and the complete bioinformatic analysis. RR constructed the Pyrococcus deletion strain. IW did the *in vitro* transcription and the footprinting experiments. VN and LK performed the gel shift assays, KB the ChIP-seq experiments and the qPCR assays. MK, NW, ZE, MM, and CZ did the negative stain imaging. FG, DG, and WH wrote the manuscript and DG and WH coordinated and supervised the work. All authors agreed to the final version of the manuscript.

## Conflict of Interest

The authors declare that the research was conducted in the absence of any commercial or financial relationships that could be construed as a potential conflict of interest.

## Footnotes

1 www.kfb-regensburg.de

2 ftp://ftp.ncbi.nih.gov/pub/wolf/COGs/arCOG/

## References

[B1] Agarwal S. Hong D. Desai N. K. Sazinsky M. H. Argüello J. M. Rosenzweig A. C. (2010). Structure and interactions of the C-terminal metal binding domain of *Archaeoglobus fulgidus* CopA. *Prot. Struct. Funct. Bioinform.* 78 2450–2458. 10.1002/prot.22753 20602459 PMC2919055

[B2] Aparicio O. Geisberg J. V. Sekinger E. Yang A. Moqtaderi Z. Struhl K. (2005). Chromatin immunoprecipitation for determining the association of proteins with specific genomic sequences in vivo. *Curr. Protoc. Mol. Biol.* 69 21.3.1–21.3.23. 10.1002/0471142727.mb2103s69 18265358

[B3] Argüello J. M. (2003). Identification of ion-selectivity determinants in heavy-metal transport P1B-type ATPases. *J. Membr. Biol.* 195 93–108. 10.1007/s00232-003-2048-2 14692449

[B4] Bailey T. L. Boden M. Buske F. A. Frith M. Grant C. E. Clementi L. (2009). MEME SUITE: tools for motif discovery and searching. *Nucl. Acids Res.* 37 W202–W208. 10.1093/nar/gkp335 19458158 PMC2703892

[B5] Baker-Austin C. Dopson M. Wexler M. Sawers R. G. Bond P. L. (2005). Molecular insight into extreme copper resistance in the extremophilic archaeon *Ferroplasma acidarmanus* Fer1. *Microbiology* 151 2637–2646. 10.1099/mic.0.28076-0 16079342

[B6] Bini E. (2010). Archaeal transformation of metals in the environment. *FEMS Microbiol. Ecol.* 73 1–16. 10.1111/j.1574-6941.2010.00876.x 20455933

[B7] Blombach F. Smollett K. L. Grohmann D. Werner F. (2016). Molecular mechanisms of transcription initiation — structure. function, and evolution of tfe / tfiie-like factors and open complex formation. *J. Mol. Biol.* 428 2592–2606. 10.1016/j.jmb.2016.04.016 27107643 PMC7616663

[B8] Bolger A. M. Lohse M. Usadel B. (2014). Trimmomatic: a flexible trimmer for Illumina sequence data. *Bioinformatics* 30 2114–2120. 10.1093/bioinformatics/btu170 24695404 PMC4103590

[B9] Brukner I. Sánchez R. Suck D. Pongor S. (1995). Sequence-dependent bending propensity of DNA as revealed by DNase I: parameters for trinucleotides. *EMBO J.* 14 1812–1818. 10.1002/j.1460-2075.1995.tb07169.x 7737131 PMC398274

[B10] Chen S. Iannolo M. Calvo J. M. (2005). Cooperative binding of the leucine-responsive regulatory protein (Lrp) to DNA. *J. Mol. Biol.* 345 251–264. 10.1016/j.jmb.2004.10.047 15571719

[B11] Dahlke I. (2002). A *Pyrococcus* homolog of the leucine-responsive regulatory protein. LrpA, inhibits transcription by abrogating RNA polymerase recruitment. *Nucl. Acids Res.* 30 701–710. 10.1093/nar/30.3.701 11809882 PMC100285

[B12] Darnell C. L. Tonner P. D. Gulli J. G. Schmidler S. C. Schmid A. K. (2017). Systematic discovery of archaeal transcription factor functions in regulatory networks through quantitative phenotyping analysis. *mSystems* 2 1–20. 10.1128/msystems.00032-17 28951888 PMC5605881

[B13] de los Rios S. Perona J. J. (2007). Structure of the *Escherichia coli* Leucine-responsive Regulatory Protein Lrp reveals a novel octameric assembly. *J. Mol. Biol.* 366 1589–1602. 10.1016/j.jmb.2006.12.032 17223133 PMC1933502

[B14] Denis A. Martínez-Núñez M. A. Tenorio-Salgado S. Perez-Rueda E. (2018). Dissecting the Repertoire of DNA-Binding Transcription Factors of the Archaeon *Pyrococcus furiosus* DSM 3638. *Life* 8:40. 10.3390/life8040040 30248960 PMC6316755

[B15] Dobin A. Davis C. A. Schlesinger F. Drenkow J. Zaleski C. Jha S. (2013). STAR: ultrafast universal RNA-seq aligner. *Bioinformatics* 29 15–21. 10.1093/bioinformatics/bts635 23104886 PMC3530905

[B16] Ettema T. J. G. Brinkman A. B. Lamers P. P. Kornet N. G. de Vos W. M. van der Oost J. (2006). Molecular characterization of a conserved archaeal copper resistance (cop) gene cluster and its copper-responsive regulator in *Sulfolobus solfataricus* P2. *Microbiology* 152 1969–1979. 10.1099/mic.0.28724-0 16804172

[B17] Ettema T. J. G. Huynen M. A. De Vos W. M. Van Der Oost J. (2003). TRASH: a novel metal-binding domain predicted to be involved in heavy-metal sensing, trafficking and resistance. *Trends Biochem. Sci.* 28 170–173. 10.1016/S0968-0004(03)00037-9 12713899

[B18] Fiala G. Stetter K. O. (1986). *Pyrococcus furiosus* sp. nov. represents a novel genus of marine heterotrophic archaebacteria growing optimally at 100°C. *Arch. Microbiol.* 145 56–61. 10.3747/pdi.2011.00058 22215659 PMC3525437

[B19] Gindner A. Hausner W. Thomm M. (2014). The TrmB family: a versatile group of transcriptional regulators in Archaea. *Extremophiles* 18 925–936. 10.1007/s00792-014-0677-2 25116054 PMC4158304

[B20] Grant C. E. Bailey T. L. Noble W. S. (2011). FIMO: scanning for occurrences of a given motif. *Bioinformatics* 27 1017–1018. 10.1093/bioinformatics/btr064 21330290 PMC3065696

[B21] Grohmann D. Werner F. (2011). Recent advances in the understanding of archaeal transcription. *Curr. Opin. Microbiol.* 14 328–334. 10.1016/j.mib.2011.04.012 21596617

[B22] Grünberger F. Reichelt R. Bunk B. Spröer C. Overmann J. Rachel R. (2019). Next Generation DNA-Seq and Differential RNA-Seq allow Re-annotation of the *Pyrococcus furiosus* DSM 3638 genome and provide insights into archaeal antisense transcription. *Front. Microbiol.* 10:01603. 10.3389/fmicb.2019.01603 31354685 PMC6640164

[B23] Grünberger F. Reichelt R. Waege I. Ned V. Bronner K. Kaljanac M. (2020). CopR, a global regulator of transcription to maintain copper homeostasis in *Pyrococcus furiosus*. *bioRxiv[Preprint].* 10.1101/2020.08.14.251413 PMC7830388 33505379

[B24] Gunther M. R. Hanna P. M. Mason R. P. Cohen M. S. (1995). Hydroxyl radical formation from cuprous ion and hydrogen peroxide: a spin-trapping study. *Arch. Biochem. Biophys.* 316 515–522. 10.1006/abbi.1995.1068 7840659

[B25] Honarmand Ebrahimi K. Hagedoorn P. L. Hagen W. R. (2015). Unity in the biochemistry of the iron-storage proteins ferritin and bacterioferritin. *Chem. Rev.* 115 295–326. 10.1021/cr5004908 25418839

[B26] Hong S. K. Jeong J. Sung M. K. Kang G. Kim M. Ran A. (2019). Characterization of the copper-sensing transcriptional regulator CopR from the hyperthermophilic archeaon *Thermococcus onnurineus* NA1. *BioMetals* 2 1–15. 10.1007/s10534-019-00223-2 31676935

[B27] Huber R. Burggraf S. Mayer T. Barns S. M. Rossnagel P. Stetter K. O. (1995). Isolation of a hyperthermophilic archaeum predicted by in situ RNA analysis. *Nature* 376 57–58. 10.1038/376057a0 7541115

[B28] Karr E. A. (2014). Transcription Regulation in the Third Domain. *Adv. Appl. Microbiol.* 89 101–133. 10.1016/B978-0-12-800259-9.00003-2 25131401

[B29] Kaur A. Pan M. Meislin M. Facciotti M. T. El-gewely R. Baliga N. S. (2006). A systems view of haloarchaeal strategies to withstand stress from transition metals A systems view of haloarchaeal strategies to withstand stress from transition metals. *Genome Res.* 16 841–854. 10.1101/gr.5189606 16751342 PMC1484451

[B30] Khatibi P. A. Chou C. J. Loder A. J. Zurawski J. V. Adams M. W. W. Kelly R. M. (2017). Impact of growth mode, phase, and rate on the metabolic state of the extremely thermophilic archaeon *Pyrococcus furiosus*. *Biotechnol. Bioeng.* 114 2947–2954. 10.1002/bit.26408 28840937 PMC5809124

[B31] Kopylova E. Noé L. Touzet H. (2012). SortMeRNA: fast and accurate filtering of ribosomal RNAs in metatranscriptomic data. *Bioinformatics* 28 3211–3217. 10.1093/bioinformatics/bts611 23071270

[B32] Kreuzer M. Schmutzler K. Waege I. Thomm M. Hausner W. (2013). Genetic engineering of *Pyrococcus furiosus* to use chitin as a carbon source. *BMC Biotechnol.* 13:9. 10.1186/1472-6750-13-9 23391022 PMC3575233

[B33] Kumarevel T. Nakano N. Ponnuraj K. Gopinath S. C. B. Sakamoto K. Shinkai A. (2008). Crystal structure of glutamine receptor protein from *Sulfolobus tokodaii* strain 7 in complex with its effector l -glutamine: implications of effector binding in molecular association and DNA binding. *Nucl. Acids Res.* 36 4808–4820. 10.1093/nar/gkn456 18653535 PMC2504300

[B34] Lagorce A. Fourçans A. Dutertre M. Bouyssiere B. Zivanovic Y. Confalonieri F. (2012). Genome-wide transcriptional response of the Archaeon *Thermococcus gammatolerans* to Cadmium. *PLoS One* 7:e0041935. 10.1371/journal.pone.0041935 22848664 PMC3407056

[B35] Langmead B. Salzberg S. L. (2012). Fast gapped-read alignment with Bowtie 2. *Nat. Methods* 9 357–359. 10.1038/nmeth.1923 22388286 PMC3322381

[B36] Leleu M. Lefebvre G. Rougemont J. (2010). Processing and analyzing ChIP-seq data: from short reads to regulatory interactions. *Brief. Funct. Genomics* 9 466–476. 10.1093/bfgp/elq022 20861161 PMC3080774

[B37] Lemmens L. Maklad H. R. Bervoets I. Peeters E. (2019). Transcription regulators in archaea: homologies and differences with bacterial regulators. *J. Mol. Biol.* 431 4132–4146. 10.1016/j.jmb.2019.05.045 31195017

[B38] Leonard P. M. Smits S. H. J. Sedelnikova S. E. Brinkman A. B. De Vos W. M. Van Der Oost J. (2001). Crystal structure of the Lrp-like transcriptional regulator from the archaeon *Pyrococcus furiosus*. *EMBO J.* 20 990–997. 10.1093/emboj/20.5.990 11230123 PMC145483

[B39] Li H. Handsaker B. Wysoker A. Fennell T. Ruan J. Homer N. (2009). The Sequence Alignment/Map format and SAMtools. *Bioinformatics* 25 2078–2079. 10.1093/bioinformatics/btp352 19505943 PMC2723002

[B40] Liao Y. Smyth G. K. Shi W. (2019). The R package Rsubread is easier, faster, cheaper and better for alignment and quantification of RNA sequencing reads. *Nucl. Acids Res.* 47 e47–e47. 10.1093/nar/gkz114 30783653 PMC6486549

[B41] Lipscomb G. L. Keese A. M. Cowart D. M. Schut G. J. Thomm M. Adams M. W. W. (2009). SurR: a transcriptional activator and repressor controlling hydrogen and elemental sulphur metabolism in *Pyrococcus furiosus*. *Mol. Microbiol.* 71 332–349. 10.1111/j.1365-2958.2008.06525.x 19017274 PMC2745277

[B42] Lipscomb G. L. Stirrett K. Schut G. J. Yang F. Jenney F. E. Scott R. A. (2011). Natural competence in the hyperthermophilic archaeon *Pyrococcus furiosus* facilitates genetic manipulation: construction of markerless deletions of genes encoding the two cytoplasmic hydrogenases. *Appl. Environ. Microbiol.* 77 2232–2238. 10.1128/AEM.02624-10 21317259 PMC3067412

[B43] Liu H. Orell A. Maes D. van Wolferen M. Ann-Christin L. Bernander R. (2014). BarR, an Lrp-type transcription factor in *Sulfolobus acidocaldarius*, regulates an aminotransferase gene in a β-alanine responsive manner. *Mol. Microbiol.* 92 625–639. 10.1111/mmi.12583 24646198

[B44] Love M. I. Huber W. Anders S. (2014). Moderated estimation of fold change and dispersion for RNA-seq data with DESeq2. *Genome Biol.* 15:550. 10.1186/s13059-014-0550-8 25516281 PMC4302049

[B45] Macomber L. Imlay J. A. (2009). The iron-sulfur clusters of dehydratases are primary intracellular targets of copper toxicity. *Proc. Natl. Acad. Sci. U. S. A.* 106 8344–8349. 10.1073/pnas.0812808106 19416816 PMC2688863

[B46] Makarova K. Wolf Y. Koonin E. (2015). Archaeal Clusters of Orthologous Genes (arCOGs): an update and application for analysis of shared features between thermococcales. methanococcales, and methanobacteriales. *Life* 5 818–840. 10.3390/life5010818 25764277 PMC4390880

[B47] Mana-Capelli S. Mandal A. K. Argüello J. M. (2003). *Archaeoglobus fulgidus* CopB is a thermophilic Cu2+-ATPase: functional role of its histidine-rich N-terminal metal binding domain. *J. Biol. Chem.* 278 40534–40541. 10.1074/jbc.M306907200 12876283

[B48] Mandal A. K. Cheung W. D. Argüello J. M. (2002). Characterization of a thermophilic P-type Ag+/Cu+-ATPase from the extremophile *Archaeoglobus fulgidus*. *J. Biol. Chem.* 277 7201–7218. 10.1074/jbc.M109964200 11756450

[B49] Martell D. J. Joshi C. P. Gaballa A. Santiago A. G. Chen T.-Y. Jung W. (2015). Metalloregulator CueR biases RNA polymerase’s kinetic sampling of dead-end or open complex to repress or activate transcription. *Proc. Natl. Acad. Sci. U.S.A.* 112 13467–13472. 10.1073/pnas.1515231112 26483469 PMC4640755

[B50] Martínez-Bussenius C. Navarro C. A. Jerez C. A. (2017). Microbial copper resistance: importance in biohydrometallurgy. *Microb. Biotechnol.* 10 279–295. 10.1111/1751-7915.12450 27790868 PMC5328820

[B51] Meloni G. Zhang L. Rees D. C. (2014). Transmembrane type-2-like Cu2+ site in the P1B-3-type ATPase CopB: implications for metal selectivity. *ACS Chem. Biol.* 9 116–121. 10.1021/cb400603t 24144006 PMC3947036

[B52] Meysman P. Collado-Vides J. Morett E. Viola R. Engelen K. Laukens K. (2014). Structural properties of prokaryotic promoter regions correlate with functional features. *PLoS One* 9:e0088717. 10.1371/journal.pone.0088717 24516674 PMC3918002

[B53] Nickels P. C. Wünsch B. Holzmeister P. Bae W. Kneer L. M. Grohmann D. (2016). Molecular force spectroscopy with a DNA origami-based nanoscopic force clamp. *Science* 354 305–307. 10.1126/science.aah5974 27846560 PMC6546592

[B54] Ochs S. M. Thumann S. Richau R. Weirauch M. T. Lowe T. M. Thomm M. (2012). Activation of Archaeal Transcription Mediated by Recruitment of Transcription Factor B. *J. Biol. Chem.* 287 18863–18871. 10.1074/jbc.M112.365742 22496454 PMC3365709

[B55] Orell A. Remonsellez F. Arancibia R. Jerez C. A. (2013). Molecular characterization of copper and cadmium resistance determinants in the biomining thermoacidophilic archaeon *Sulfolobus metallicus*. *Archaea* 2013:289236. 10.1155/2013/289236 23509422 PMC3595675

[B56] Ouhammouch M. (2001). A thermostable platform for transcriptional regulation: the DNA-binding properties of two Lrp homologs from the hyperthermophilic archaeon *Methanococcus jannaschii*. *EMBO J.* 20 146–156. 10.1093/emboj/20.1.146 11226165 PMC140199

[B57] Ouhammouch M. Dewhurst R. E. Hausner W. Thomm M. Geiduschek E. P. (2003). Activation of archaeal transcription by recruitment of the TATA-binding protein. *Proc. Natl. Acad. Sci. U.S.A.* 100 5097–5102. 10.1073/pnas.0837150100 12692306 PMC154304

[B58] Ouhammouch M. Langham G. E. Hausner W. Simpson A. J. El-Sayed N. M. A. Geiduschek E. P. (2005). Promoter architecture and response to a positive regulator of archaeal transcription. *Mol. Microbiol.* 56 625–637. 10.1111/j.1365-2958.2005.04563.x 15819620

[B59] Peeters E. Albers S.-V. Vassart A. Driessen A. J. M. Charlier D. (2009). Ss-LrpB, a transcriptional regulator from *Sulfolobus solfataricus*, regulates a gene cluster with a pyruvate ferredoxin oxidoreductase-encoding operon and permease genes. *Mol. Microbiol.* 71 972–988. 10.1111/j.1365-2958.2008.06578.x 19170871

[B60] Peeters E. Charlier D. (2010). The Lrp family of transcription regulators in Archaea. *Archaea* 2010 1–10. 10.1155/2010/750457 21151646 PMC2995911

[B61] Peeters E. Peixeiro N. Sezonov G. (2013). Cis-regulatory logic in archaeal transcription. *Biochem. Soc. Trans.* 41 326–331. 10.1042/BST20120312 23356306

[B62] Peeters E. Thia-Toong T.-L. Gigot D. Maes D. Charlier D. (2004). Ss-LrpB, a novel Lrp-like regulator of *Sulfolobus solfataricus* P2, binds cooperatively to three conserved targets in its own control region. *Mol. Microbiol.* 54 321–336. 10.1111/j.1365-2958.2004.04274.x 15469506

[B63] Pham A. N. Xing G. Miller C. J. Waite T. D. (2013). Fenton-like copper redox chemistry revisited: hydrogen peroxide and superoxide mediation of copper-catalyzed oxidant production. *J. Catal.* 2013 54–64. 10.1016/j.jcat.2013.01.025

[B64] Philips S. J. Canalizo-Hernandez M. Yildirim I. Schatz G. C. Mondragon A. O’Halloran T. V. (2015). Allosteric transcriptional regulation via changes in the overall topology of the core promoter. *Science* 349 877–881. 10.1126/science.aaa9809 26293965 PMC4617686

[B65] Plaisier C. L. Lo F.-Y. Ashworth J. Brooks A. N. Beer K. D. Kaur A. (2014). Evolution of context dependent regulation by expansion of feast/famine regulatory proteins. *BMC Syst. Biol.* 8:122. 10.1186/s12918-014-0122-2 25394904 PMC4236453

[B66] Purohit R. Ross M. O. Batelu S. Kusowski A. Stemmler T. L. Hoffman B. M. (2018). Cu+-specific CopB transporter: revising p1B-type ATPase classification. *Proc. Natl. Acad. Sci. U.S.A.* 115 2108–2113. 10.1073/pnas.1721783115 29440418 PMC5834730

[B67] R Foundation for Statistical Computing (2018). *R: A Language and Environment for Statistical Computing.* Vienna: R Foundation for Statistical Computing.

[B68] Reichelt R. Gindner A. Thomm M. Hausner W. (2016). Genome-wide binding analysis of the transcriptional regulator TrmBL1 in *Pyrococcus furiosus*. *BMC Genomics* 17:40. 10.1186/s12864-015-2360-0 26747700 PMC4706686

[B69] Reichelt R. Ruperti K. M. A. A. Kreuzer M. Dexl S. Thomm M. Hausner W. (2018). The transcriptional regulator TFB-RF1 activates transcription of a putative ABC transporter in *Pyrococcus furiosus*. *Front. Microbiol.* 9:838. 10.3389/fmicb.2018.00838 29760686 PMC5937170

[B70] Rensing C. McDevitt S. F. (2013). The copper metallome in prokaryotic cells. *Met. Ions Life Sci.* 12 417–450. 10.1007/978-94-007-5561-1_12 23595679

[B71] Rivero M. Torres-Paris C. Muñoz R. Cabrera R. Navarro C. A. Jerez C. A. (2018). Inorganic polyphosphate, exopolyphosphatase, and Pho84 -like transporters May be involved in copper resistance in *Metallosphaera sedula* DSM 5348 T. *Archaea* 2018 1–12. 10.1155/2018/5251061 29692683 PMC5859850

[B72] Rohou A. Grigorieff N. (2015). CTFFIND4: fast and accurate defocus estimation from electron micrographs. *J. Struct. Biol.* 192 216–221. 10.1016/j.jsb.2015.08.008 26278980 PMC6760662

[B73] Santangelo T. J. Čuboňová L. Reeve J. N. (2010). *Thermococcus kodakarensis* Genetics: Tk1827-encoded β-glycosidase, new positive-selection protocol, and targeted and repetitive deletion technology. *Appl. Environ. Microbiol.* 76 1044–1052. 10.1128/AEM.02497-09 20023088 PMC2820962

[B74] Sazinsky M. H. LeMoine B. Orofino M. Davydov R. Bencze K. Z. Stemmler T. L. (2007). Characterization and structure of a Zn2+ and [2Fe-2S]-containing copper chaperone from *Archaeoglobus fulgidus*. *J. Biol. Chem.* 282 25950–25959. 10.1074/jbc.M703311200 17609202 PMC2859431

[B75] Sitsel O. Grønberg C. Autzen H. E. Wang K. Meloni G. Nissen P. (2015). Structure and function of Cu(I)- and Zn(II)-ATPases. *Biochemistry* 54 5673–5683. 10.1021/acs.biochem.5b00512 26132333

[B76] Soto D. F. Recalde A. Orell A. Albers S.-V. V. Paradela A. Navarro C. A. (2019). Global effect of the lack of inorganic polyphosphate in the extremophilic archaeon *Sulfolobus solfataricus*: a proteomic approach. *J. Proteomics* 191 143–152. 10.1016/j.jprot.2018.02.024 29501848

[B77] Strand K. R. Sun C. Li T. Jenney F. E. Schut G. J. Adams M. W. W. (2010). Oxidative stress protection and the repair response to hydrogen peroxide in the hyperthermophilic archaeon *Pyrococcus furiosus* and in related species. *Arch. Microbiol.* 192 447–459. 10.1007/s00203-010-0570-z 20379702

[B78] Tan G. Yang J. Li T. Zhao J. Sun S. Li X. (2017). Anaerobic copper toxicity and iron-sulfur cluster biogenesis in *Escherichia coli*. *Appl. Environ. Microbiol.* 83 e000867-17. 10.1128/AEM.00867-17 28576762 PMC5541227

[B79] Thorgersen M. P. Stirrett K. Scott R. A. Adams M. W. W. (2012). Mechanism of oxygen detoxification by the surprisingly oxygen-tolerant hyperthermophilic archaeon. *Pyrococcus furiosus*. *Proc. Natl. Acad. Sci. U.S.A.* 109 18547–18552. 10.1073/pnas.1208605109 23093671 PMC3494905

[B80] Torarinsson E. Klenk H. P. Garrett R. A. (2005). Divergent transcriptional and translational signals in Archaea. *Environ. Microbiol.* 7 47–54. 10.1111/j.1462-2920.2004.00674.x 15643935

[B81] Tsuda T. Toyoshima C. (2009). Nucleotide recognition by CopA, a Cu -transporting P-type ATPase. *EMBO J.* 4:9. 10.1038/emboj.2009.143 19478797 PMC2699366

[B82] Vierke G. Engelmann A. Hebbeln C. Thomm M. (2003). A novel archaeal transcriptional regulator of heat shock response. *J. Biol. Chem.* 278 18–26. 10.1074/jbc.M209250200 12381724

[B83] Villafane A. Voskoboynik Y. Cuebas M. Ruhl I. Bini E. (2009). Response to excess copper in the hyperthermophile *Sulfolobus solfataricus* strain 98/2. *Biochem. Biophys. Res. Commun.* 385 67–71. 10.1126/scisignal.2001449.Engineering 19427833 PMC2810495

[B84] Villafane A. Voskoboynik Y. Ruhl I. Sannino D. Maezato Y. Blum P. (2011). CopR of *Sulfolobus solfataricus* represents a novel class of archaeal-specific copper-responsive activators of transcription. *Microbiology* 157 2808–2817. 10.1099/mic.0.051862-0 21757491

[B85] Waege I. Schmid G. Thumann S. Thomm M. Hausner W. (2010). Shuttle vector-based transformation system for *Pyrococcus furiosus*. *Appl. Environ. Microbiol.* 76 3308–3313. 10.1128/AEM.01951-09 20363792 PMC2869139

[B86] Werner F. Grohmann D. (2011). Evolution of multisubunit RNA polymerases in the three domains of life. *Nat. Rev. Microbiol.* 9 85–98. 10.1038/nrmicro2507 21233849

[B87] Wheaton G. H. Mukherjee A. Kelly R. M. (2016). Transcriptomes of the extremely thermoacidophilic archaeon *Metallosphaera Sedula* exposed to metal “shock” reveal generic and specific metal responses. *Appl. Environ. Microbiol.* 82 4613–4627. 10.1128/AEM.01176-16 27208114 PMC4984275

[B88] Yamada M. Ishijima S. A. Suzuki M. (2009). Interactions between the archaeal transcription repressor FL11 and its coregulators lysine and arginine. *Proteins Struct. Funct. Bioinforma.* 74 520–525. 10.1002/prot.22269 19004003

[B89] Yang H. Lipscomb G. L. Keese A. M. Schut G. J. Thomm M. Adams M. W. W. (2010). SurR regulates hydrogen production in *Pyrococcus furiosus* by a sulfur-dependent redox switch. *Mol. Microbiol.* 77 1111–1122. 10.1111/j.1365-2958.2010.07275.x 20598080 PMC2975895

[B90] Yokoyama K. Ishijima S. A. Koike H. Kurihara C. Shimowasa A. Kabasawa M. (2007). Feast/famine regulation by transcription Factor FL11 for the survival of the hyperthermophilic archaeon pyrococcus OT3. *Structure* 15 1542–1554. 10.1016/j.str.2007.10.015 18073105

[B91] Young M. D. Wakefield M. J. Smyth G. K. Oshlack A. (2010). Gene ontology analysis for RNA-seq: accounting for selection bias. *Genome Biol.* 11:R14. 10.1186/gb-2010-11-2-r14 20132535 PMC2872874

[B92] Zhu A. Ibrahim J. G. Love M. I. (2019). Heavy-tailed prior distributions for sequence count data: removing the noise and preserving large differences. *Bioinformatics* 35 2084–2092. 10.1093/bioinformatics/bty895 30395178 PMC6581436

[B93] Zhu Y. Kumar S. Menon A. L. Scott R. A. Adams M. W. W. W. (2013). Regulation of iron metabolism by *pyrococcus furiosus*. *J. Bacteriol.* 195 2400–2407. 10.1128/JB.02280-12 23504018 PMC3650548

[B94] Zivanov J. Nakane T. Forsberg B. O. Kimanius D. Hagen W. J. H. Lindahl E. (2018). New tools for automated high-resolution cryo-EM structure determination in RELION-3. *eLife* 7:e42166. 10.7554/eLife.42166 30412051 PMC6250425

