# Supplementary material for: CopR, a Global Regulator of Transcription to Maintain Copper Homeostasis in Pyrococcus furiosus
Source: Front Microbiol. 2021 Jan 11;11:613532. doi: 10.3389/fmicb.2020.613532 (PMC7830388; doi:10.3389/fmicb.2020.613532)
Supplement: Supplementary file 4 [file Data_Sheet_1.docx]

Supplementary Material

# Supplementary Figures


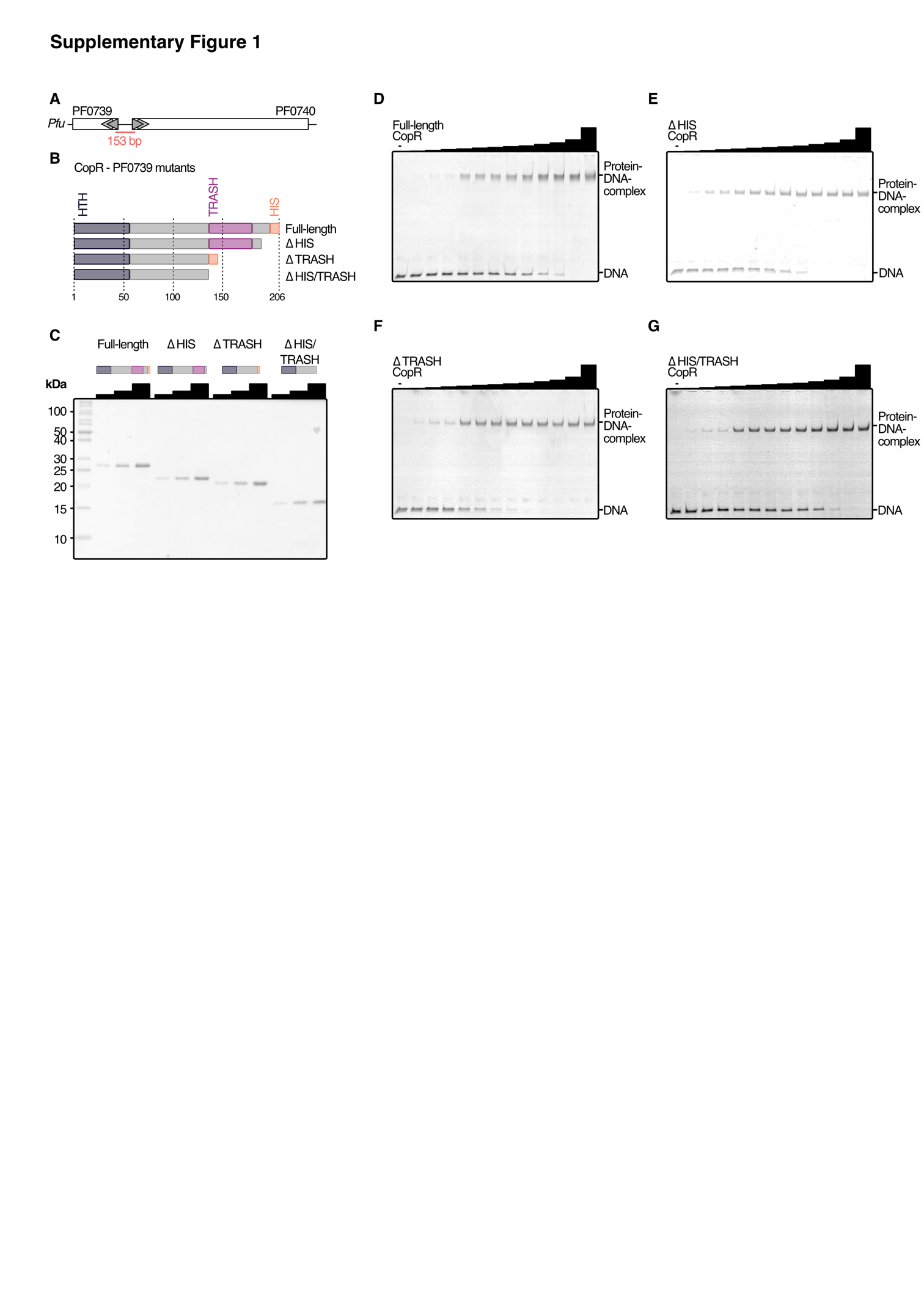


Supplementary Figure 1. CopR (PF0739) binds to the promoter region of *copA* (PF0740). A, Schematic representation of the *copR/copA* locus in *P. furiosus*. The template used for the EMSA analysis is highlighted in red and contains both translation start sites. B, Schematic of mutants generated for the functional characterisation of CopR. DNA-binding helix-turn-helix (HTH), metal-sensing TRASH domain and additional C-terminal Histidine-rich sequence are highlighted in different colors. C, SDS-PAGE analysis of purified recombinant proteins. D, EMSA analysis were performed using 20 nM of DNA (153 b, see panel a) and increasing concentrations of recombinant protein (12.5, 25, 37.5, 50, 62.5, 75, 87.5, 100, 125, 150, 200, 400 nM).


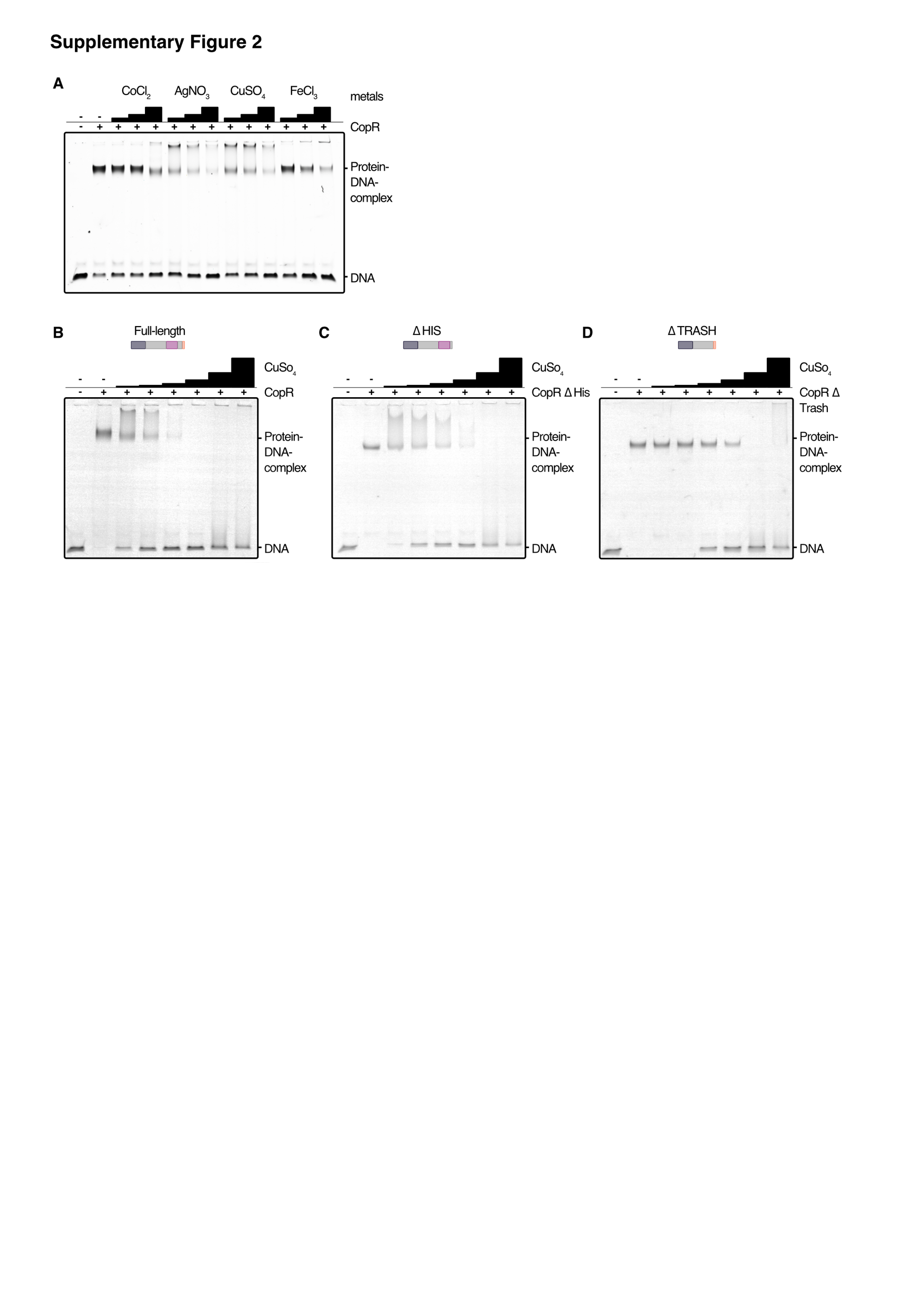


Supplementary Figure 2. Metal specificity of CopR and domain-deleted mutants in *P. furiosus*. A, EMSA analysis was performed using 20 nM DNA (*copR/copA* promoter), 200 nM full-length protein and increasing concentrations (12.5, 25, 50 µM) of the respective metal (CoCl_2_, AgNO_3_, CuSO_4_, FeCl_3_). B, Influence of increasing CuSO_4_ concentrations (50, 100, 200, 400, 800, 1600 µM) on the DNA-binding behavior of full-length CopR, C, CopR∆HIS and D, CopR∆TRASH using 20 nM DNA and 200 nM protein.


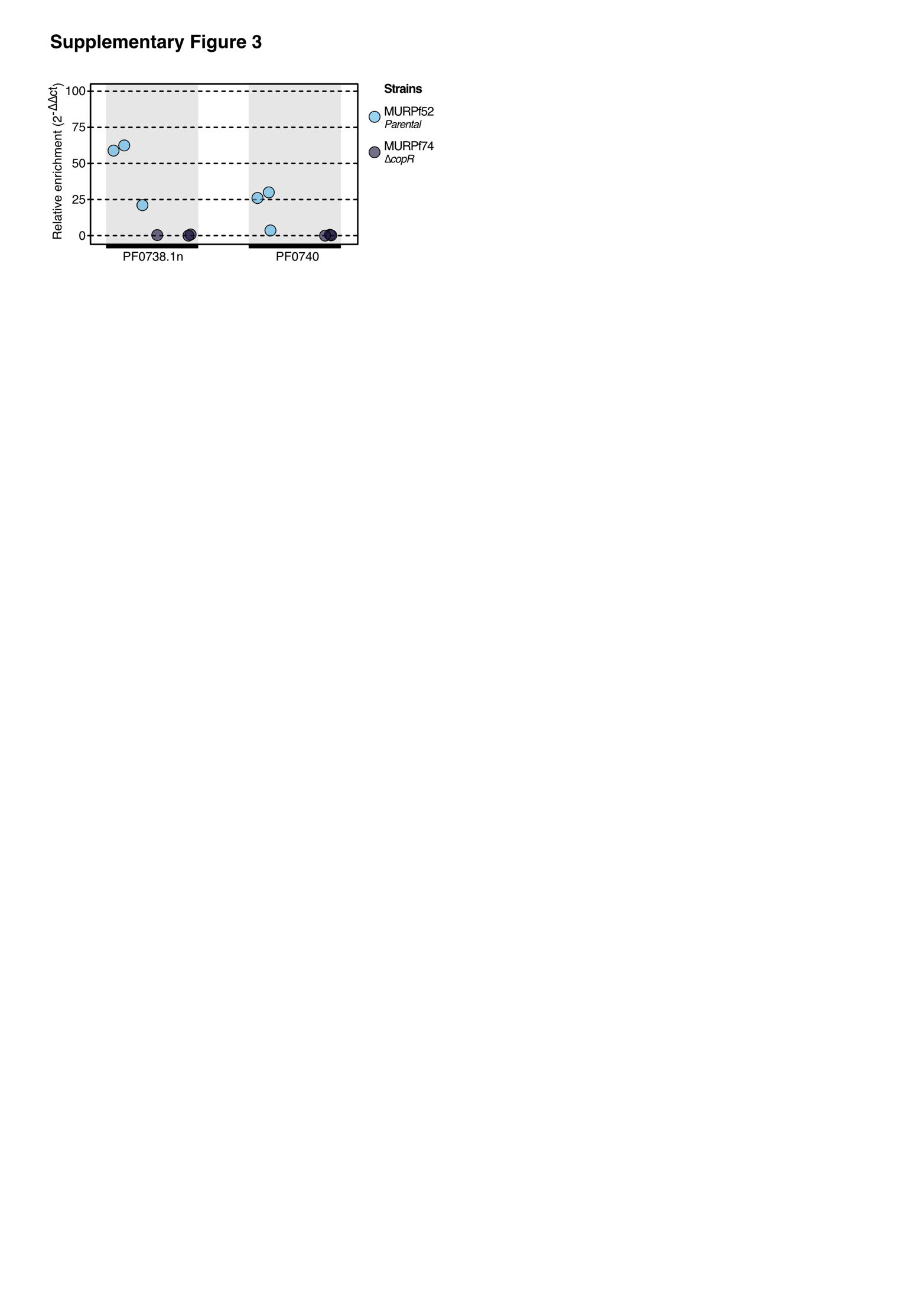


Supplementary Figure 3. Relative enrichment of *pf0740* and *pf0738.1n* measured by RT-qPCR. Expression levels from biological triplicates (individual points are shown) were compared to a house-keeping gene *pf0256* in *Pyrococcus furiosus* parental strain MURPf52 and ∆*copR* strain MURPf74. Enrichment was calculated using ∆∆ct method.


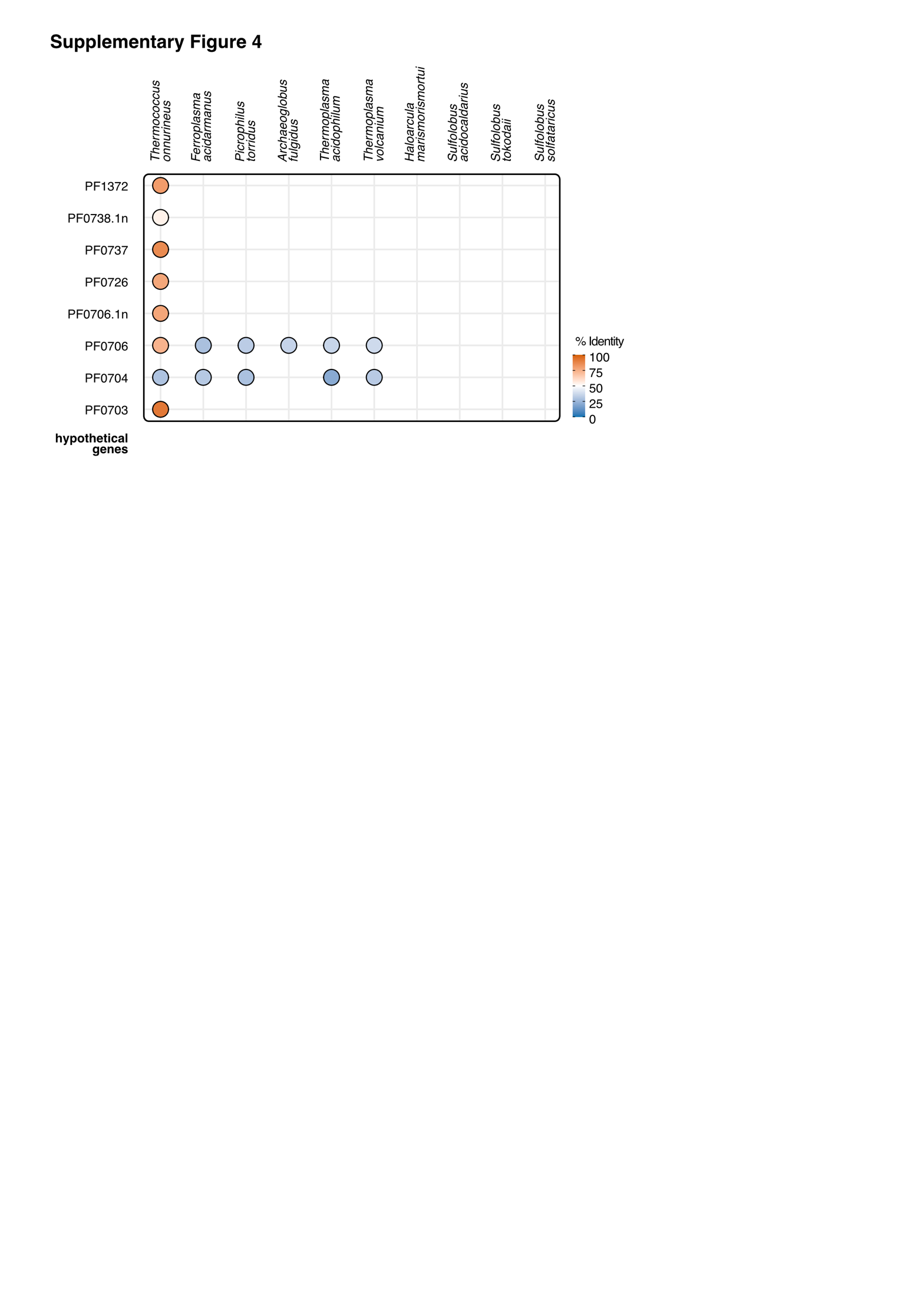


Supplementary Figure 4. Conservation of copper-induced hypothetical genes from *P. furiosus* in other cop-cluster containing archaea. BLAST (with standard parameters) was used to detect homologous protein sequences in selected archaea. Sequence identity is indicated by the color scale.


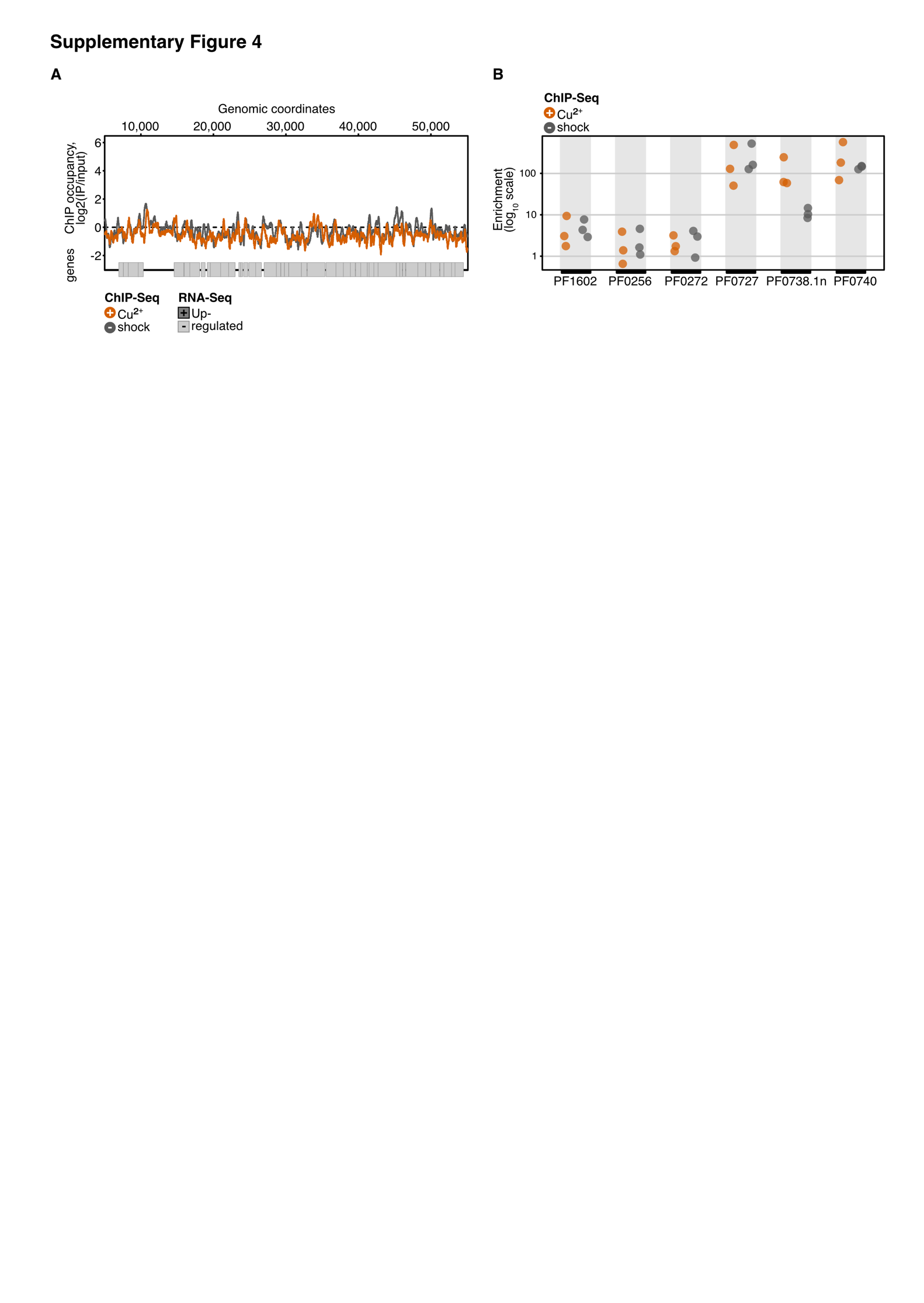


Supplementary Figure 5. Confirmatory analysis of ChIP-seq results. A, Exemplary CopR-unbound 50-kb region of *P. furiosus* (compare Fig. 4A). ChIP-seq curves were generated for Cu^2+^ shocked (orange) and untreated (grey) samples by comparing the IPs to input samples (mean values of triplicates are shown). Genome annotation is shown at the bottom according to scale with significantly up-regulated genes (adjusted p value < 0.05, Log_2_ fold change +/- Cu^2+^ > 1) colored in dark grey. B, ChIP-qPCR results of multiple ChIP-seq identified CopR-unbound (PF1602, PF0256, PF0272) and CopR-bound (PF0727, PF0738.1n, PF0740) regions. Data are shown as individual points of biological replicates for normal conditions (grey) and copper-treated cells (orange). Values are calculated as fold enrichment over input sample.


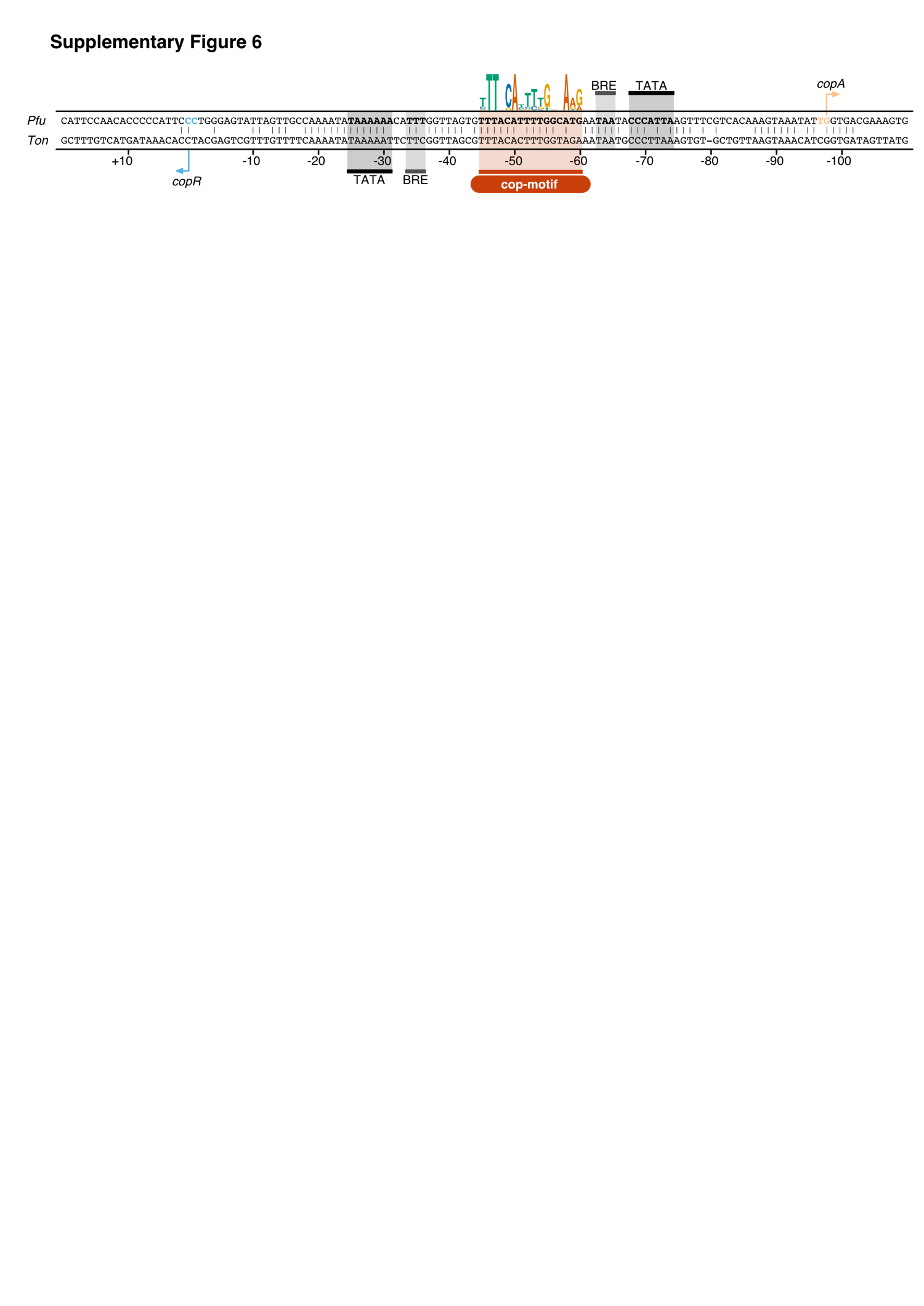


Supplementary Figure 6. Pairwise sequence alignment of CopR/CopA promoter regions in *P. furiosus* and *T. onnurineus*. Regions of high similarity were identified using BLAST, matches are highlighted by connected lines, while gaps are indicated by a minus-sign within the sequence. Promoter elements, transcription start sites (TSS) and the CopR recognition motif are highlighted in the *P. furiosus* sequence. Distance is calculated dependent from Pfu-CopR TSS.


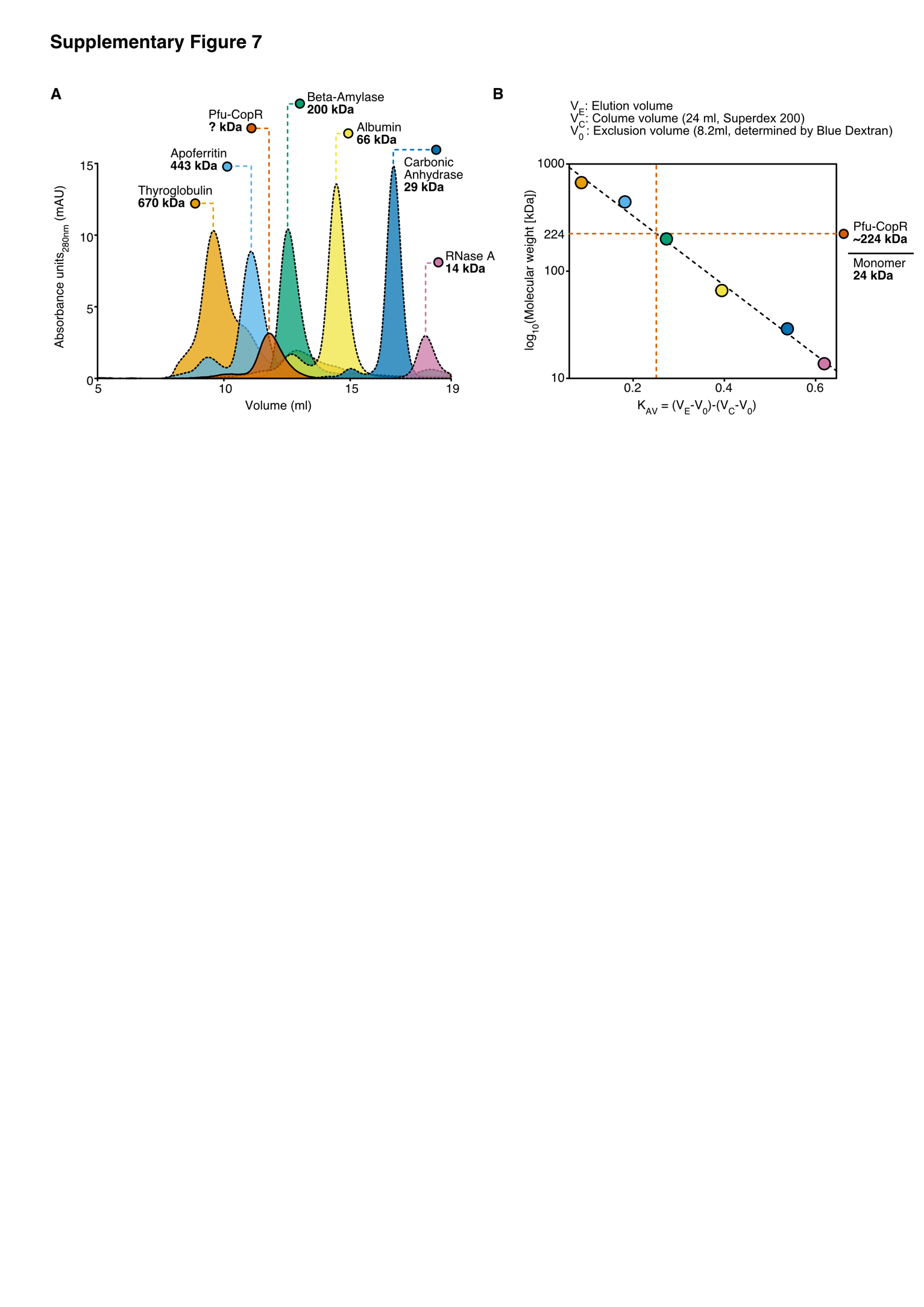


Supplementary Figure 7. Size-exclusion chromatography analysis of CopR. A, Gel filtration chromatograms of selected standards and CopR from *P. furiosus*. B, Calibration curve for oligomeric state estimation of CopR. The curve used to estimate the native molecular weight based on the elution position during analytical gel filtration of CopR is indicated by a black dashed line.

# Supplementary Tables

**Supplementary Table 1.** Used strains, plasmids and primer sequences.

**Supplementary Table 2.** Illumina sequencing and mapping statistics. Table summarizing the sequencing and mapping statistics after RNA sequencing (total number of reads), rRNA removal using SortMeRNA (Kopylova et al., 2012), trimming using trimmomatic (Bolger et al., 2014) and mapping using STAR (Dobin et al., 2013). Note that rRNA-derived reads have not been excluded from the ChIP-seq analysis in sheet 2.

**Supplementary Table 3.** DGE results from DESeq2. Table displaying the results from the differential gene expression analysis performed using DESeq2 (Love et al., 2014). *Pyrococcus* gene identifiers are given for the old and new annotation (Grünberger et al., 2019), including the gene annotation and arCOG classification (Makarova et al., 2015). The mean counts column is based on the count values of biological triplicates from normal and copper shock conditions. Positive fold changes indicate an up regulation upon copper treatment.

References

Bolger, A. M., Lohse, M., and Usadel, B. (2014). Trimmomatic: A flexible trimmer for Illumina sequence data. *Bioinformatics*. doi:10.1093/bioinformatics/btu170.

Dobin, A., Davis, C. A., Schlesinger, F., Drenkow, J., Zaleski, C., Jha, S., et al. (2013). STAR: Ultrafast universal RNA-seq aligner. *Bioinformatics* 29, 15–21. doi:10.1093/bioinformatics/bts635.

Grünberger, F., Reichelt, R., Bunk, B., Spröer, C., Overmann, J., Rachel, R., et al. (2019). Next Generation DNA-Seq and Differential RNA-Seq Allow Re-annotation of the Pyrococcus furiosus DSM 3638 Genome and Provide Insights Into Archaeal Antisense Transcription. *Front. Microbiol.* 10. doi:10.3389/fmicb.2019.01603.

Kopylova, E., Noé, L., and Touzet, H. (2012). SortMeRNA: Fast and accurate filtering of ribosomal RNAs in metatranscriptomic data. *Bioinformatics* 28, 3211–3217. doi:10.1093/bioinformatics/bts611.

Love, M. I., Huber, W., and Anders, S. (2014). Moderated estimation of fold change and dispersion for RNA-seq data with DESeq2. *Genome Biol.* 15, 550. doi:10.1186/s13059-014-0550-8.

Makarova, K., Wolf, Y., and Koonin, E. (2015). Archaeal Clusters of Orthologous Genes (arCOGs): An Update and Application for Analysis of Shared Features between Thermococcales, Methanococcales, and Methanobacteriales. *Life* 5, 818–840. doi:10.3390/life5010818.
